# Serotonin and neuropeptides are both released by the HSN command neuron to initiate *Caenorhabditis elegans* egg laying

**DOI:** 10.1371/journal.pgen.1007896

**Published:** 2019-01-24

**Authors:** Jacob C. Brewer, Andrew C. Olson, Kevin M. Collins, Michael R. Koelle

**Affiliations:** 1 Department of Molecular Biophysics & Biochemistry, Yale University, New Haven, Connecticut, United States of America; 2 Department of Biology, University of Miami, Coral Gables, Florida, United States of America; Brown University, UNITED STATES

## Abstract

Neurons typically release both a small-molecule neurotransmitter and one or more neuropeptides, but how these two types of signal from the same neuron might act together remains largely obscure. For example, serotonergic neurons in mammalian brain express the neuropeptide Substance P, but it is unclear how this co-released neuropeptide might modulate serotonin signaling. We studied this issue in *C*. *elegans*, in which all serotonergic neurons express the neuropeptide NLP-3. The serotonergic Hermaphrodite Specific Neurons (HSNs) are command motor neurons within the egg-laying circuit which have been shown to release serotonin to initiate egg-laying behavior. We found that egg-laying defects in animals lacking serotonin were far milder than in animals lacking HSNs, suggesting that HSNs must release other signal(s) in addition to serotonin to stimulate egg laying. While null mutants for *nlp-3* had only mild egg-laying defects, animals lacking both serotonin and NLP-3 had severe defects, similar to those of animals lacking HSNs. Optogenetic activation of HSNs induced egg laying in wild-type animals, and in mutant animals lacking either serotonin or NLP-3, but failed to induce egg laying in animals lacking both. We recorded calcium activity in the egg-laying muscles of animals lacking either serotonin, NLP-3, or both. The single mutants, and to a greater extent the double mutant, showed muscle activity that was uncoordinated and unable to expel eggs. Specifically, the vm2 muscles cells, which are direct postsynaptic targets of the HSN, failed to contract simultaneously with other egg-laying muscle cells. Our results show that the HSN neurons use serotonin and the neuropeptide NLP-3 as partially redundant co-transmitters that together stimulate and coordinate activity of the target cells onto which they are released.

## Author summary

Activity of the brain results from neurons communicating with each other using chemical signals. A typical neuron releases two kinds of chemical signals: a small molecule neurotransmitter, such as serotonin, and one or more small proteins, called neuropeptides. For example, neurons in the human brain that release serotonin, a neurotransmitter thought to be involved in depression, also release the neuropeptide Substance P. Neuroscientists have typically studied the effects of neurotransmitters and neuropeptides separately, without considering how these two types of signals from the same neuron might be integrated. Here we analyzed how specific neurons in the model organism *C*. *elegans* use both serotonin and a neuropeptide together. The Hermaphrodite Specific Neurons (HSNs) activate a small group of neurons and muscles to generate egg-laying behavior. Killing the HSNs resulted in animals unable to lay eggs, but we found that eliminating either serotonin or the neuropeptide resulted in HSNs that still remained able to activate egg laying. However, eliminating both serotonin and the neuropeptide resulted in HSNs unable to activate coordinated contractions of the egg-laying muscles. Our results show that in a living animal, serotonin acts in concert with a co-released neuropeptide to carry out its functions.

## Introduction

Drugs that selectively manipulate serotonin signaling are widely used to treat depression and other psychiatric disorders, yet these drugs are often ineffective, and no specific molecular defects in serotonin signaling have been identified as the cause of these disorders [1]. This situation suggests there is more to understand about the basic science of serotonin signaling that could help explain the cause of psychiatric disorders. One feature of serotonin signaling in the mammalian brain that remains poorly understood is that serotonin neurons appear to also release a specific neuropeptide, Substance P [2–6]. Of the ~80 billion neurons in the human brain, only about 100,000 make serotonin. Their cell bodies are concentrated in the dorsal raphe nuclei of the brain stem, but they extend axons throughout the brain that release serotonin to influence many brain functions [2,7,8]. Several methods have been used to measure the proportion of serotonin neurons that express Substance P in the various raphe subnuclei of human or rat brain, with results suggesting that somewhere between 25–100% of serotonin neurons also express substance P [2,5,9,10]. The apparent co-release of serotonin and Substance P from the same neurons is one instance of the broad but poorly studied phenomenon of co-transmission by small-molecule neurotransmitters and neuropeptides [11–14]. It remains unclear how exactly co-released serotonin and neuropeptide(s) might functionally interact. Clinical studies of Substance P antagonists showed that they, like selective serotonin reuptake inhibitors, can have significant anti-depressant activity [15–17]. One study in the brain stem respiratory circuit indicated that serotonin and substance P each independently stimulate activity of the circuit [10], but the complexity of mammalian brain circuits makes precise analysis of such effects difficult.

Small neural circuits of invertebrates provide the potential for more precise analysis of the functional effects of co-transmission. In these circuits, every cell can be identified, the small-molecule neurotransmitter and neuropeptide content of each cell can be determined, and the functional effects of each signal can potentially be characterized. An elegant body of work on small circuits from crustaceans has used pharmacological and electrophysiological methods to analyze the functional effects of co-transmitters [11]. However, the nature of these experimental systems typically requires that the isolated circuit be studied after dissection from the animal and often precludes genetic tests of transmitter function.

*C*. *elegans* provides the opportunity to use powerful genetic methods to functionally analyze co-transmission within small circuits of intact, freely-behaving animals. Mutations and transgenes can be used to manipulate neurotransmitters, neuropeptides, and their receptors, optogenetic methods can be used to manipulate activity of presynaptic cells, and the functional consequences of all these manipulations on postsynpatic cells can be read out using genetically-encoded Ca^2+^ indicators and by measuring animal behavior. Despite the promise of this approach, there are so far few examples of its use to study co-transmission [18,19].

Here, we have applied the genetic toolbox described above to analyze the functional consequences of co-transmission by serotonin and a neuropeptide within a well-characterized small circuit of *C*. *elegans*. The *C*. *elegans* egg-laying circuit contains three neuron types that signal each other and the egg-laying muscles to generate ~2 minute active phases, during which rhythmic circuit activity induces egg-laying behavior, that alternate with ~20 minute inactive phases, during which the circuit is largely silent and no eggs are laid [20]. The two serotonergic hermaphrodite specific neurons [HSNs) serve as the command neurons [21] within this circuit in that 1) worms lacking HSNs are egg-laying defective [22]; 2) optogenetic activation of HSNs is sufficient to induce activity of the circuit that mimics a spontaneous active phase [23–26]; and 3) no other cells in the circuit have these properties [23]. We show here that the HSNs use a combination of serotonin and a neuropeptide to induce the coordinated circuit activity of egg-laying active phases.

## Results

### The serotonergic HSN egg-laying neurons remain largely functional without serotonin

**Fig 1:**
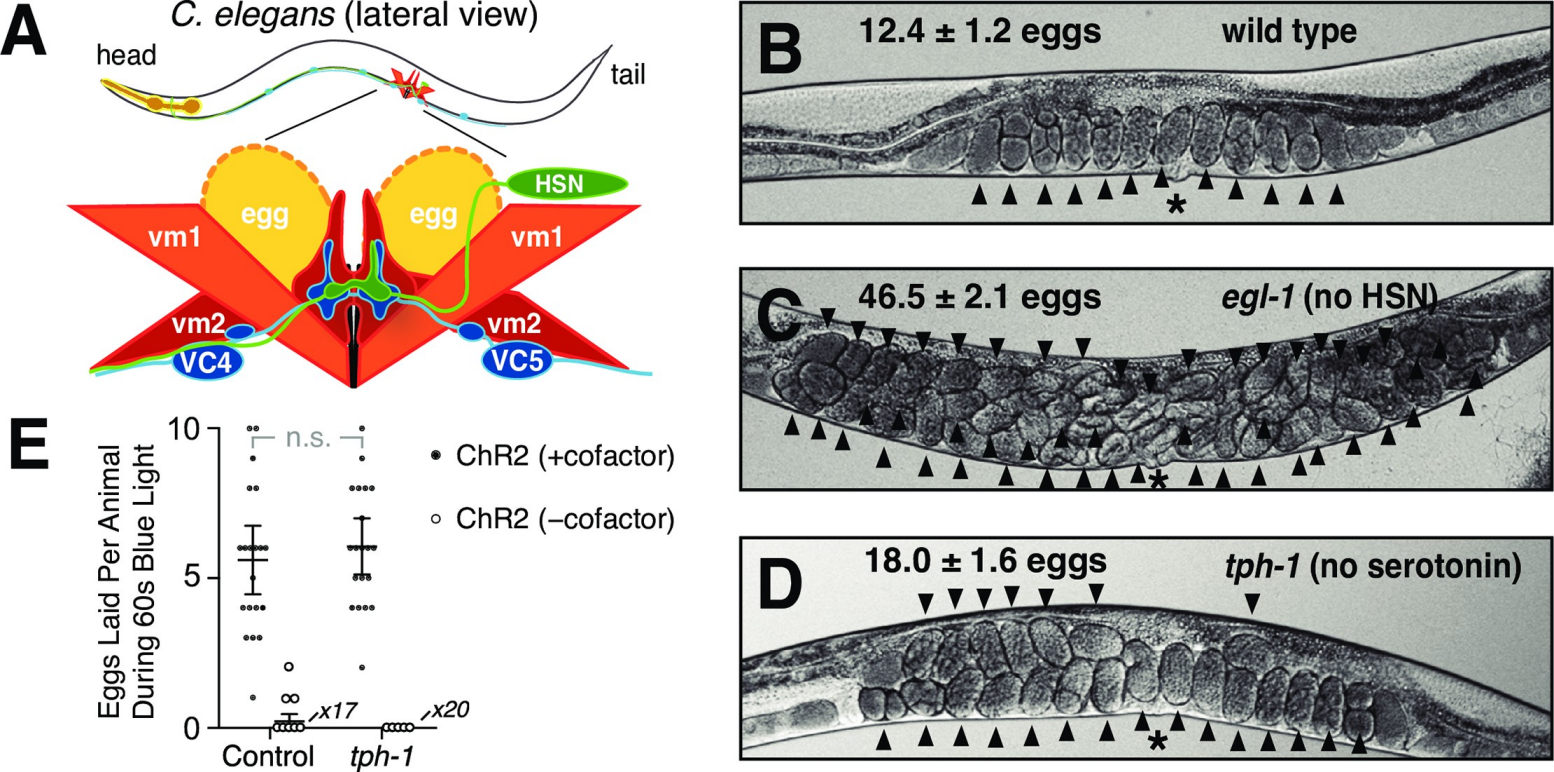
Serotonin is not required for the HSN to stimulate egg laying. **A)** Schematic of the *C*. *elegans* egg-laying circuit, adapted from [23]. HSN and VC motorneurons synapse onto vm2 vulval muscle cells, which along with vm1 muscle cells contract to open the vulva and release eggs. Only the left HSN and vulval muscle cells are shown–equivalent cells are also found on the right side of the animal. The uv1 neuroendocrine cells that inhibit the circuit are not shown. **B-D)** Images of representative animals of the indicated genotypes, showing the average number of unlaid eggs +/- 95% confidence intervals, n = 30. Arrowheads indicate individual unlaid eggs. Asterisks indicate the location of the vulva. **E)** Number of eggs laid during 60 seconds of blue light exposure by animals expressing ChR2 in the HSNs. Both control and *tph-1* animals also carry *lite-1(ce314)* mutations that eliminate a locomotion response to blue light [69]. Black filled data points, animals that were grown for a generation in the presence of ChR2’s required cofactor all-trans retinal (ATR). Open circle data points, negative control animals grown in the absence of ATR. For this and all subsequent graphs: error bars represent 95% confidence intervals for the mean unless otherwise indicated; n.s. indicates no statistically significant difference (p>0.05); the number measurements of zero eggs for some genotypes is indicated at the horizontal axis.

The small circuit that initiates egg-laying behavior is schematized in Fig 1A. The serotonergic Hermaphrodite-Specific Neurons (HSNs), along with the cholinergic Ventral Cord type C neurons (VCs), synapse onto the type 2 vulval muscles (vm2), which contract with the type 1 vulval muscles (vm1) to expel eggs [20]. Loss of the HSNs results in a severe egg-laying defect: a mutation in *egl-1* causes death of the HSNs and results in animals that, despite continuing to make eggs, rarely lay them [27], resulting in the striking phenotype of adult worms bloated with accumulated unlaid eggs (Fig 1B and 1C). Because addition of exogenous serotonin to worm culture media is sufficient to induce egg laying, even in worms lacking HSNs [22], it has been suggested that HSNs induce circuit activity simply by releasing serotonin, and thereby sensitizing the egg-laying muscles to activation by the acetylcholine released by other motor neurons of the circuit [23,28].

Contrary to this model, we found that animals lacking serotonin had only mild egg-laying defects (Fig 1D). The *tph-1* gene encodes the serotonin biosynthetic enzyme tryptophan hydroxylase, and animals with a *tph-1* null mutation have no serotonin detectable by anti-serotonin antibodies or HPLC analysis [29,30]. *tph-1* mutant animals had only mild egg-laying defects (~18 unlaid eggs), appearing more similar to the wild type (~12 unlaid eggs) than they did to *egl-1* mutants lacking HSNs (~47 unlaid eggs; Fig 1B–1D). This result, along with previous pharmacological, genetic, and behavioral studies of the function of serotonin in egg laying [31], is consistent with the idea that serotonin release can only partially explain how the HSNs initiate egg laying.

To determine definitively if serotonin is required for the HSNs to stimulate egg laying, we optogenetically stimulated the HSNs of animals either wild-type for *tph-1* or deleted for the *tph-1* gene (Fig 1E). Animals with channelrhodopsin (ChR2) expressed in the HSNs and that are wild-type for *tph-1* have been shown previously to lay eggs within a few seconds of exposure to blue light, but only if the required ChR2 cofactor all-trans retinal (ATR) is supplied to the worms [24,25]. We found that upon optogenetic activation of HSNs, *tph-1* mutant animals laid a number of eggs statistically indistinguishable from the number laid by control animals wild-type for *tph-*1 (Fig 1E and S1 Video). Thus the HSNs do not require serotonin to stimulate egg-laying behavior.

### The neuropeptide gene *nlp-3* stimulates egg laying, and loss of both *nlp-3* and serotonin together severely reduces egg laying

Our results, along with previous studies [31–34], lead to the hypothesis that the HSNs release a co-transmitter that allows them to stimulate egg laying even without serotonin. We hypothesized that this co-transmitter could be one or more of the neuropeptides encoded by five neuropeptide genes previously shown to be expressed in the HSNs [35–37]. To test this idea we created five types of transgenic worm strains, each overexpressing one of these neuropeptide genes under its own promoter. Each strain had an extrachromosomal transgene composed of multiple copies of a ~45 kb *C*. *elegans* genomic DNA clone containing one of the neuropeptide genes. Overexpressing a neuropeptide gene in this way can result in a gain-of-function phenotype caused by an increase in the normal signaling effects of the encoded neuropeptides [19,38]. Therefore, for a neuropeptide that induces egg laying, we expected overexpression to cause an increase in the frequency of egg-laying behavior.

**Fig 2:**
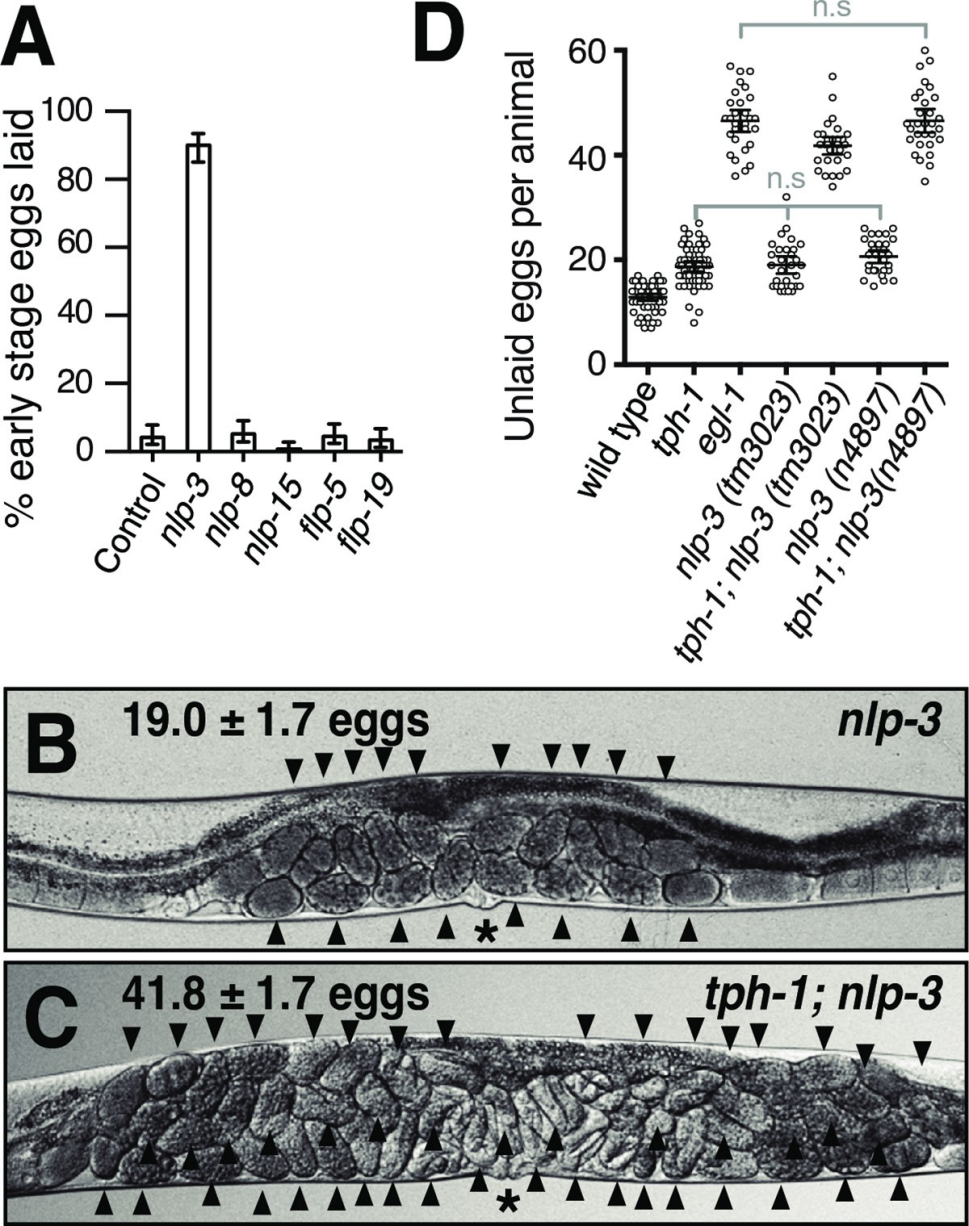
The neuropeptide gene *nlp-3*, together with serotonin, stimulates egg laying. **A)** Overexpression of *nlp-3*, but not of four other neuropeptide genes, increased the rate of egg-laying behavior. Genomic clones for each neuropeptide gene, or the coinjection marker alone (control), were injected into *C*. *elegans* to generate high-copy extrachromosomal transgenes. For each gene, 250 freshly laid eggs (50 from each of five independent transgenic lines) were examined and the percent laid at early stages of development (eight cells or fewer) was determined. Only *nlp-3* overexpression gave results significantly different from the control (p<0.05). **B-C)** Representative images of *nlp-3* and *tph-1; nlp-3* animals showing the average number of unlaid eggs, n = 30. **D)** Graph of the number of unlaid eggs for the strains indicated. Two independent deletion alleles of *nlp-3* were used. n≥30 for each strain. Comparisons labeled n.s. showed no significant differences (p>0.05). All other pairwise comparisons were significantly different with p<0.033.

Indeed, we found that overexpressing the *nlp-3* neuropeptide gene resulted in a dramatic increase in the frequency of egg-laying behavior. An increased rate of egg-laying behavior results in a decrease in the time eggs have in the mother to develop before they are laid [39]. More than 80% of the eggs laid by worms carrying the high-copy *nlp-3* transgene were laid at early stages of development, compared to ~5% for control animals not overexpressing any neuropeptide (Fig 2A). Overexpressing any of the other four neuropeptide genes did not increase the frequency of egg laying (Fig 2A).

We obtained *nlp-3* null mutant animals in which the *nlp-3* gene is deleted. Unlike the *egl-1* mutants lacking HSNs that accumulate ~47 unlaid eggs (Fig 1C), *nlp-3* null mutants accumulated only ~19 unlaid eggs (Fig 2B), and thus were more similar to the wild type (Fig 1B) or *tph-1* mutant worms lacking serotonin (Fig 1D). However, when we made *tph-1; nlp-3* double mutants so that the HSNs lacked both serotonin and NLP-3 neuropeptides, the adult animals were bloated with ~42 unlaid eggs and thus showed a severe egg-laying defect similar to that of *egl-1* animals. We obtained a second, independent deletion mutant for *nlp-3* and observed the same mild defect in the single mutant and a similar severe defect in the double mutant with *tph-1* (Fig 2D).

### The HSN egg-laying neurons use both serotonin and NLP-3 neuropeptides to stimulate egg laying

**Fig 3:**
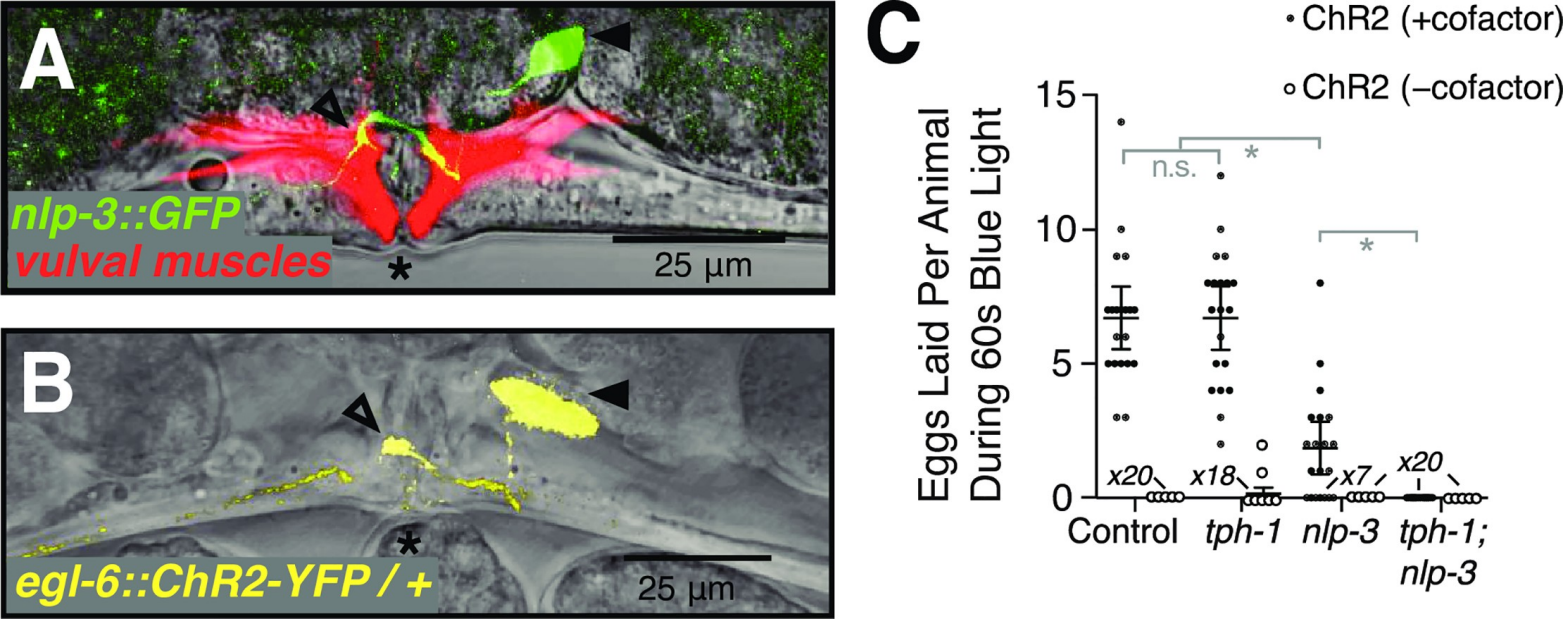
The HSNs require the *nlp-3* neuropeptide gene and *tph-1* to stimulate egg laying. **A)** *nlp-3* is expressed specifically in the HSNs. Vulval region of an adult animal carrying an *nlp-3*::GFP transgene and a second transgene that expresses mCherry in the vulval muscles from the *unc-103e* promoter [47]. **B)** The *egl-6p*::ChR2::YFP transgene is expressed specifically in the HSN. In A and B, asterisks indicate the vulva, filled arrowheads the HSN cell body and open arrowheads the HSN synapse onto the vulval muscles. **C)** Average number of eggs laid during 60 seconds of blue light exposure by animals heterozygous for the *egl-6p*::ChR2::YFP transgene and homozygous for the indicated null mutations in *tph-1* and/or *nlp-3*. Control, animals wild-type for *tph-1* and *nlp-3*. Black filled data points, animals grown in the presence of ChR2’s required cofactor all-trans retinal (ATR). Open circle data points, animals grown in the absence of ATR. All animals in this experiment were homozygous for a *lite-1* mutation that abolished an endogenous *C*. *elegans* response to blue light [64]. Error bars, 95% confidence intervals of the mean. The number of measurements of zero eggs for some genotypes is indicated at the horizontal axis. Key pairwise comparisons are indicated (n.s., not significantly different, p>0.05; or *, significantly different, p<0.001).

The above experiments demonstrated that serotonin and NLP-3 stimulate egg laying but did not examine if they do so by being released from the HSN neurons. Previous studies demonstrated that the HSNs contain serotonin, and it was inferred from indirect evidence that HSNs can release serotonin to stimulate egg laying [22,40,41]. We used the *nlp-3* promoter to drive GFP expression and saw, as previously reported [42], that *nlp-3* is expressed in the HSN neurons but no other cells of the egg-laying circuit (Fig 3A), consistent with the hypothesis that NLP-3 neuropeptides are released from HSNs to stimulate egg laying.

To directly test what combination of transmitters the HSNs use to stimulate egg laying, we generated animals that express ChR2::YFP in the HSN neurons (Fig 3B) and (as controls) that were wild-type for *tph-1* and *nlp-3*, or that carried null mutations in *tph-1*, *nlp-3*, or both. We then tested whether optogenetic stimulation of the HSNs could induce egg laying. Both the control animals and null mutants for *tph-1* laid eggs readily upon ChR2 activation, with no statistically significant differences in the number of eggs laid (Fig 3C) or in several other measures of the egg-laying behavior induced (e.g. time to first egg laid, time to last egg laid, S2A–S2C Fig). However, whereas all wild-type and *tph-1* animals tested laid eggs upon ChR2 activation, 7/20 *nlp-3* mutant animals failed to lay any eggs, and the 13/20 that did lay eggs laid fewer on average than did the wild-type or *tph-1* animals. No eggs were laid by any *tph-1; nlp-3* double mutant animals (Fig 3C). Therefore, we conclude that: 1) the HSN neurons release both serotonin and NLP-3 peptides to stimulate egg laying; 2) either signal alone is sufficient to stimulate at least some egg laying; and 3) when lacking both signals the HSNs have no detectable ability to stimulate the behavior.

### NLP-3 release from cells other than HSN neurons has some ability to stimulate egg laying

**Fig 4:**
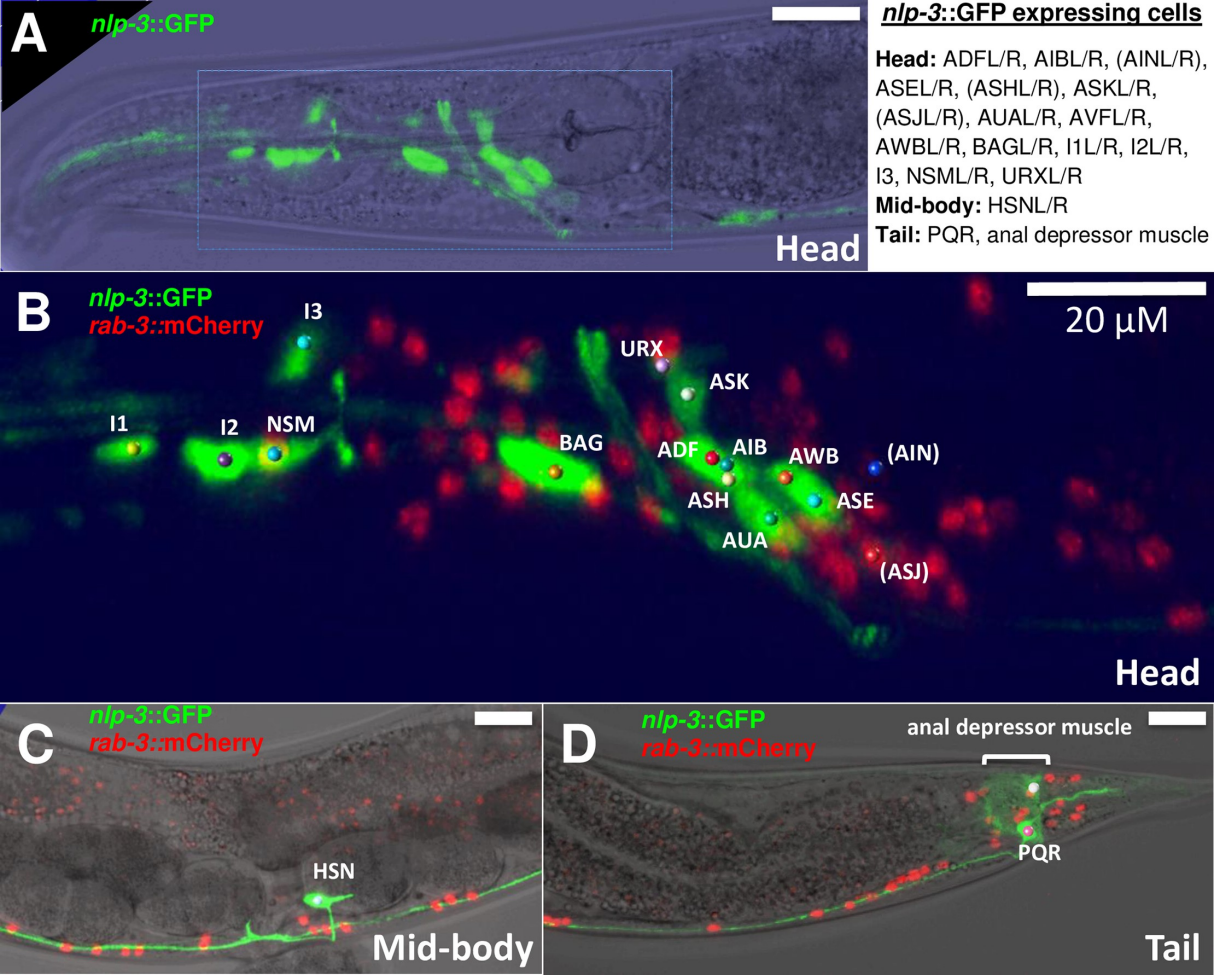
Expression pattern of *nlp-3*. The *nlp-3* promoter was used to drive expression of GFP in transgenic animals. Animals also express nuclear-localized tagRFP in all neurons from the *rab-3* promoter. **A)** *nlp-3*::GFP fluorescence superimposed on a bright-field image of the head. **B)** The area outlined by the rectangle in A is enlarged, and both GFP and tagRFP fluorescence are shown. Colored balls indicate cell bodies of GFP-expressing neurons, and are labeled with their cell designations. **C)** and **D)** Show midbody and tail regions, respectively, with GFP and mCherry fluorescence superimposed on bright-field images, with GFP-expressing cells identified. All images show the left side of the animal only and all scale bars are 20 μm. A list of all cells that express *nlp-3* is shown at upper right. AIN and ASJ are not visibly GFP labeled in the image shown, and along with ASH are more weakly labeled than other *nlp-3-*expressing cells. The AVFL/R neurons are just outside of the area displayed in B but are shown in S4 Video.

While the experiments shown in Fig 3C demonstrate that NLP-3 released from the HSN stimulates egg laying, they did not test whether NLP-3 might also be released from additional cells to stimulate egg laying. To investigate this issue, we first determined the entire set of cells in *C*. *elegans* that express the *nlp-3* gene. We used a transgene to drive expression of GFP from the *nlp-3* promoter, and by combining this with red fluorescent markers of various subsets of identified neurons, identified every *nlp-3*-expressing cell (Fig 4, S3 Fig and S4 Video). These comprise 18 neuron types, including 16 bilaterally symmetric neuron pairs plus two unpaired neurons, totaling 34 neurons. Six of these neurons express *nlp-3*::GFP barely above background levels, while the other 28 gave strong GFP signals (Fig 4). One muscle cell also expresses *nlp-3*::GFP. The HSNs are the only neurons that express *nlp-3*::GFP that are in the midbody or that are known to play a role in egg laying. We note that Nathoo et al. [35] had previously identified a subset of the *nlp-3*-expressing cells, and our results largely agree with the earlier work.

**Fig 5:**
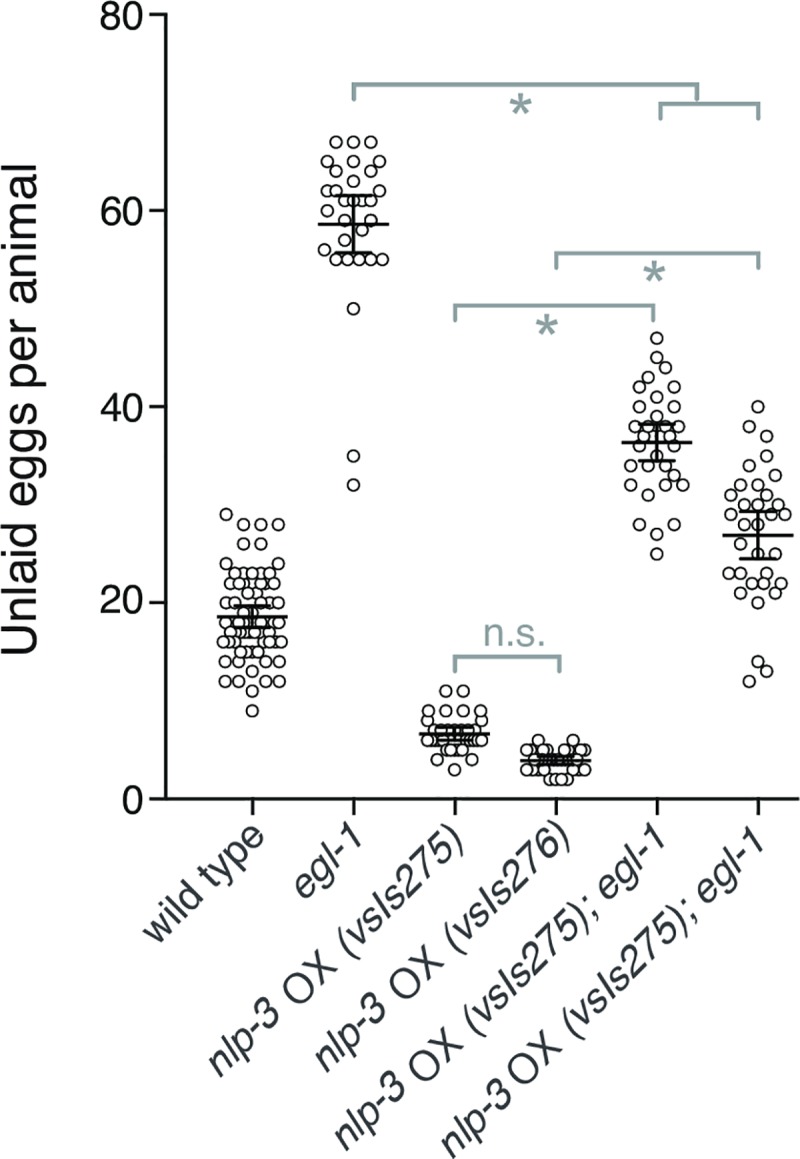
Overexpression of *nlp-3* in cells other than HSNs has some ability to stimulate egg laying. Two different chromosomally-integrated transgenes that carry multiple copies of *nlp-3* genomic DNA were used to overexpress ("OX") *nlp-3*. Egg-laying behavior was assessed by counting unlaid eggs in adult animals either carrying the overexpressor transgenes by themselves or in combination with an *egl-1* mutation that results in absence of HSN neurons. *nlp-3* overexpression significantly reduced the accumulation of eggs caused by the egl-1 mutation. n.s., no statistically significant difference (p>0.05); *, significant difference with p<0.0001.

To test if any cells besides the HSNs might release NLP-3 to stimulate egg laying, we crossed together the *egl-1* mutation (previously used in Fig 1) that results in absence of the HSNs with transgenes that overexpress *nlp-*3 from its own promoter. As seen in Fig 5, *egl-1* animals retain a large number of unlaid eggs, while animals carrying either of two independent *nlp-3* overexpressor transgenes are hyperactive egg layers and therefore retain fewer unlaid eggs than does the wild type. Animals carrying both the *egl-1* mutation and an *nlp-3* overexpressor transgene had an intermediate phenotype, retaining fewer eggs than the animals carrying the *egl-1* mutation alone and more eggs than animals carrying the *nlp-3* overexpressor alone. This result demonstrates that, at least when nlp-3 is overexpressed, release of NLP-3 from some cells other than HSN is able to stimulate egg-laying behavior to some extent.

An experiment that could help determine the extent to which NLP-3 release from the HSNs versus other cells is normally used to stimulate egg laying would be to re-express NLP-3 specifically in the HSN of *nlp-3* null mutants to determine if this is sufficient to rescue the egg-laying defects of these animals. In attempting this experiment, we found that transgenes expressed in the HSN of *nlp-3* mutants tend to cause defects in HSN development, similar to those shown in S1A Fig. This effect made the results of the rescue experiment uninterpretable. We conclude that the HSN does use NLP-3 to stimulate egg laying, and that it is possible that other NLP-3-expressing cells may also contribute to the activation of egg laying by releasing NLP-3, but the extent to which they do so remains unclear.

### Serotonin and NLP-3 can each stimulate egg laying in the absence of the other

**Fig 6:**
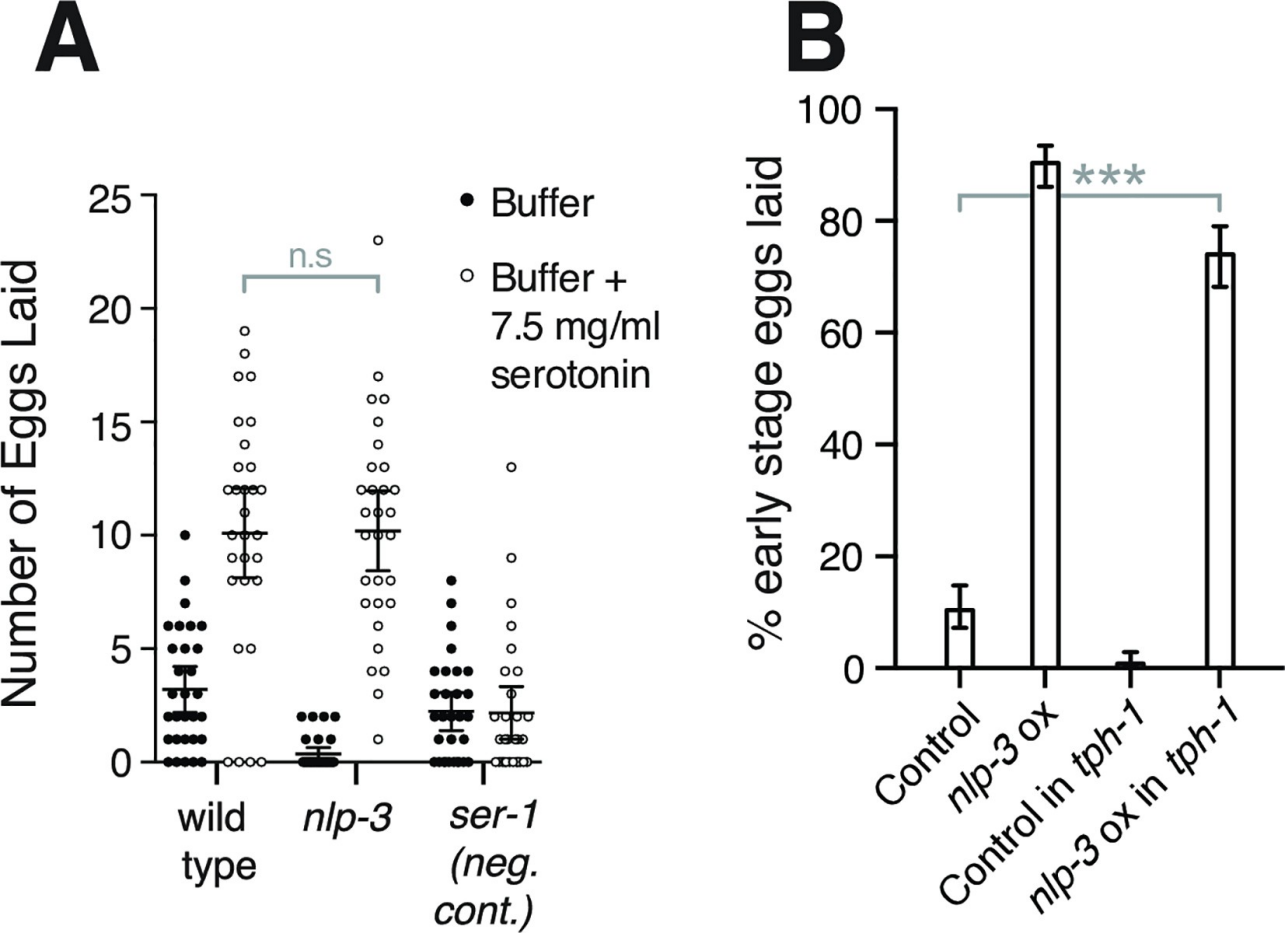
Serotonin and NLP-3 neuropeptides can stimulate egg laying in the absence of each other. **A)** Exogenous serotonin stimulates egg laying in wild-type and *nlp-3* animals. The number of eggs laid by 10 animals over 30 minutes in M9 buffer or M9 buffer plus 7.5 mg/ml serotonin was measured, and this assay was repeated >10 times per genotype. The *ser-1* serotonin receptor null mutant is the negative control. **B)** *nlp-3* overexpression stimulates egg laying even in the absence of serotonin. Animals wild-type for *tph-1* or *tph-1* null mutants were injected with marker DNA alone (control) or *nlp-3* genomic DNA plus marker DNA to overexpress *nlp-3* (*nlp-3* ox). In each case, five independent transgenic lines were produced, and 50 freshly laid eggs per line (250 eggs total per condition) were examined to determine their developmental stages. Error bars, 95% confidence intervals.

To further investigate the relationship between serotonin and NLP-3 in activating egg-laying behavior, we performed additional experiments to test if either of these transmitters is required to allow the other to stimulate egg laying. For serotonin stimulation of egg laying, we used a standard assay [22] in which worms were placed in microtiter wells with M9 buffer or M9 buffer containing 7.5 mg/ml serotonin, and the number of eggs laid in 60 minutes was counted. We saw, as observed previously [40,43–45], that exogenous serotonin stimulates egg laying in wild-type animals, but not in animals deleted for the serotonin receptor gene *ser-1* (Fig 6A). Null mutants for *nlp-3* were stimulated by serotonin to lay eggs at the same rate as were the wild-type controls, demonstrating that NLP-3 is not required for serotonin to stimulate egg laying.

We used a converse experiment to test if NLP-3 could stimulate egg-laying in the absence of serotonin. We generated *C*. *elegans* transgenes that overexpressed *nlp-3* by containing multiple copies of *nlp-3* genomic DNA or control transgenes that did not overexpress *nlp-3*. In a strain background wild-type for *tph-1*, we observed (Fig 6B), as we had seen previously in an analogous experiment (Fig 2A), that overexpression of *nlp-3* resulted in hyperactive egg laying as evidenced by a high percentage of early-stage eggs laid. When we carried out this same experiment in a *tph-1* null mutant, *nlp-3* overexpression also resulted in hyperactive egg laying, albeit at a somewhat reduced level (Fig 6B). Thus serotonin is not required to allow *nlp-3* overexpression to induce egg laying, but the absence of serotonin may mildly reduce the effects of NLP-3.

### Serotonin and NLP-3 together cause the HSN postsynaptic targets, the vm2 muscle cells, to contract coordinately with other egg-laying muscles

**Fig 7:**
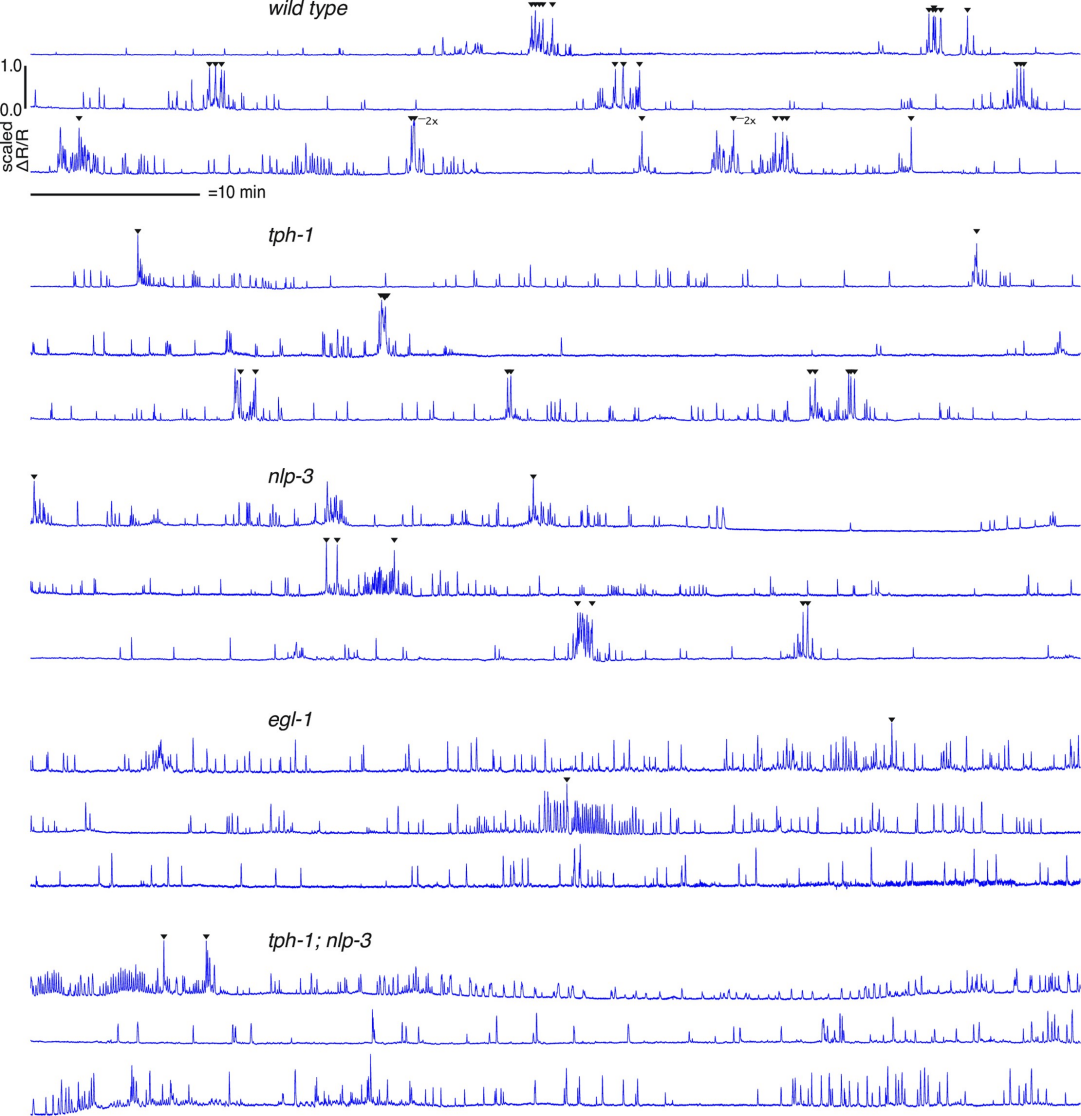
Vulval muscle activity, but not egg release, occurs frequently in mutants lacking serotonin, NLP-3, or both. Graphs of Ca^2+^ transients showing ΔR/R of GCaMP5/mCherry signal recorded over 1 hour for each of three different animals per genotype. Arrows indicate a calcium transient associated with an egg-laying event. “2x” indicates that two eggs were laid nearly simultaneously during the same calcium transient. Scale bar, 10 minutes. Vertical scales have been normalized to depict comparable peak heights in all animals shown.

To understand the functional effects of the HSN co-transmitters, we recorded Ca^2+^ activity of the vulval muscle cells, which are postsynaptic targets of the HSNs, in animals that were wild-type, lacked serotonin, lacked NLP-3, or lacked both. To do so, we co-expressed the Ca^2+^-sensitive green fluorescent protein GCaMP5 and the Ca^2+^-insensitive red fluorescent protein mCherry in the vm2 muscle cells, the direct postsynaptic targets of the HSNs, and also in the vm1 muscle cells, which are gap-junctioned to vm2 and have been thought to contract with vm2 to expel eggs [46]. Using methods we previously developed [23,47,48], we carried out ratiometric fluorescence imaging of intact animals (S2 Video) to measure Ca^2+^ transients under conditions that allow egg-laying behavior to proceed as it does in standard lab culture, such that in wild-type animals ~2 minute egg-laying active phases occur about every 20 minutes. Fig 7 shows traces of one-hour recordings of Ca^2+^ transients in the entire ensemble of vm1 and vm2 cells together for three animals of each of the following genotypes: wild-type, *tph-1* and *nlp-3* single mutants, the *tph-1; nlp-3* double mutant, and *egl-1* animals lacking HSNs.

We observed frequent vulval muscle activity in all genotypes: the recordings in Fig 7 show hundreds of Ca^2+^ transients for each genotype. However, less than 10% of the vulval muscle Ca^2+^ transients resulted in egg release in the wild type, and even fewer successful egg-laying events occurred in mutants lacking serotonin or NLP-3 (29 eggs released over three hours for the wild type, compared to 15 for *tph-1* and 9 for *nlp-3*). Activity in animals lacking both serotonin and NLP-3 neuropeptides (*tph-1; nlp-3)* or lacking HSNs (*egl-1*) was actually more frequent than in the wild type, but very rarely produced successful egg release: each genotype released just two eggs in the three hours analyzed.

**Fig 8:**
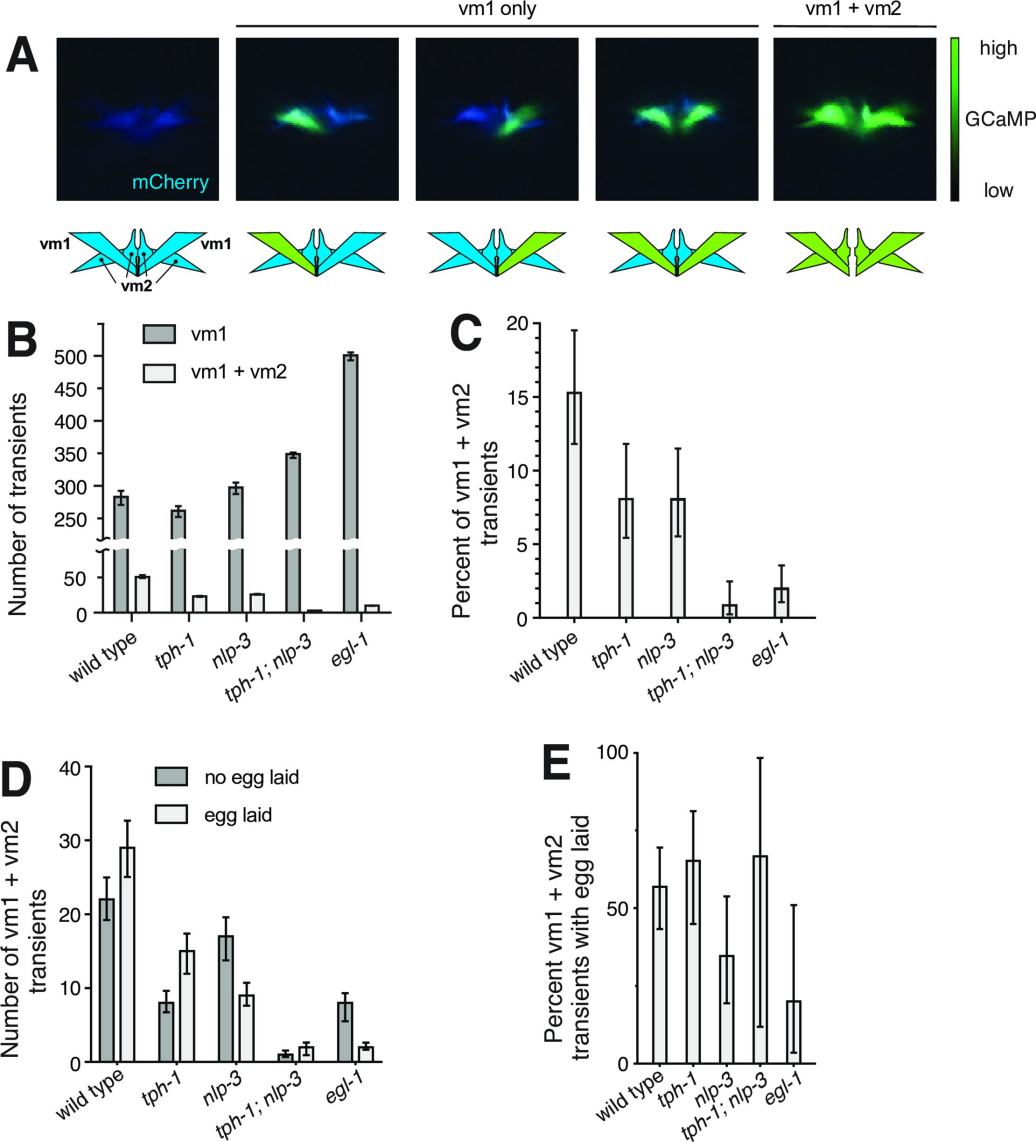
Egg-laying events are associated with vm1 + vm2 Ca^2+^ transients. **A)** Still frames from ratiometric recordings illustrating different patterns of activity observed in the vulval muscles. The mCherry channel is rendered in blue. The GCaMP channel is superimposed in green, with intensity rendered by ranging from transparent (low) to bright green (high). Schematics are shown below the images to indicate the individual muscle cells where activity appears to occur. **B-C)** A graph of the number **(B)** and percent **(C)** of calcium transients occurring in the vm1 only compared to those appearing to occur in vm1 + vm2 for each genotype. *p*-values for decreases in percentage of vm1 + vm2 transients for mutants compared to the wild type in **(C)** were = 0.05 (*tph-1*), = 0.04 (*nlp-3*), <0.001 (*tph-1; nlp-3*); and <0.001 (*egl-1*). These values indicate that *tph-1; nlp-3* and *egl-1* have significantly fewer vm1 + vm2 transients after correcting for multiple comparisons. **D-E)** The number **(D)** and proportion **(E)** of either vm1 or vm1 + vm2 transients that are associated with egg-laying events. All 57 egg-laying events occurred during vm1 + vm2 events. In **(B-E)** the data analyzed are from the three hours of recordings for each genotype depicted in Fig 7, and error bars are 95% confidence intervals for the results expected if an infinite sample size was used.

To identify the differences between vulval muscle contractions that did or did not release eggs, we adjusted how we collected images during Ca^2+^ recordings. Previously-published Ca^2+^ imaging of the vulval muscles used images focused at the center of the group of two vm1 and two vm2 muscles found on either the left or right side of the animal. The resulting images showed Ca^2+^ activity that was often found at the most ventral tip of this group of muscles, but that could not be assigned to individual muscle cells [25,46,47]. By focusing more laterally on either the left or right set of vulval muscles, we could resolve individual vm1 and vm2 cells and determine which of the four muscle cells within the set were active during any given Ca^2+^ transient detected (Fig 8A, S3 Video). All of the data presented in Fig 8 results from use of this more lateral focus.

The large majority of the muscle activity we observed in every genotype examined occurred exclusively in one or both of the vm1s imaged, with no concurrent activity detected in the vm2s, and we refer to such events as "vm1 only" (Fig 8B). We never observed an event in any genotype in which a Ca^2+^ transient occurred exclusively in vm2 cell(s) without accompanying activity in vm1 cell(s). In the wild type, ~15% of Ca^2+^ transients involved both vm1 and both vm2 cells imaged, and we refer to such events as "vm1 + vm2". In the wild type, vm1 + vm2 vulval muscle contractions occurred exclusively within active phases, the ~2-minute intervals during which eggs were laid and that contained frequent vulval muscle transients (Fig 7). Furthermore, all 29 egg release events observed in the wild type occurred during one of the 51 vm1 + vm2 vulval muscle contractions we saw during the three hours of recordings analyzed. We conclude that simultaneous contraction of all the vulval muscle cells is necessary for egg release.

Mutants lacking either serotonin, NLP-3, or both showed a similar or even greater number of vm1 Ca^2+^ transients compared to the wild type, but a decreased percent of these events were accompanied by vm2 Ca^2+^ transients to produce vm1 + vm2 events (Fig 8B and 8C). The decrease compared to the wild type in the percent of events that included vm2 was about 2-fold in *tph-1* and *nlp-3* single mutants, but 10- to 15-fold in the *tph-1; nlp-3* double mutant and in *egl-1* animals lacking HSNs. In the mutants, as in the wild-type, egg release occurred only during about half of the events that included vm2 activity (Fig 8D), with no statistically significant difference in this proportion in any of the genotypes we studied (Fig 8E). Thus Ca^2+^ activity in vm1 muscles does not require the serotonin or NLP-3 released by HSNs. However, these two signals together promote vm2 Ca^2+^ activity, and successful egg laying requires that vm1 and vm2 muscle cells contract simultaneously.

## Discussion

### HSN command neurons release serotonin and NLP-3 neuropeptides to activate and coordinate activity of the egg-laying circuit

The principal finding of this study is that the HSN command neurons release a combination of serotonin and NLP-3 peptides to activate the egg-laying circuit. Loss of either signal, and to a much greater extent, loss of both signals, results in loss of activity of the vm2 muscle cells. The vm2s are the direct postsynaptic targets of the HSNs and their activity appears to be necessary for successful release of eggs.

**Fig 9:**
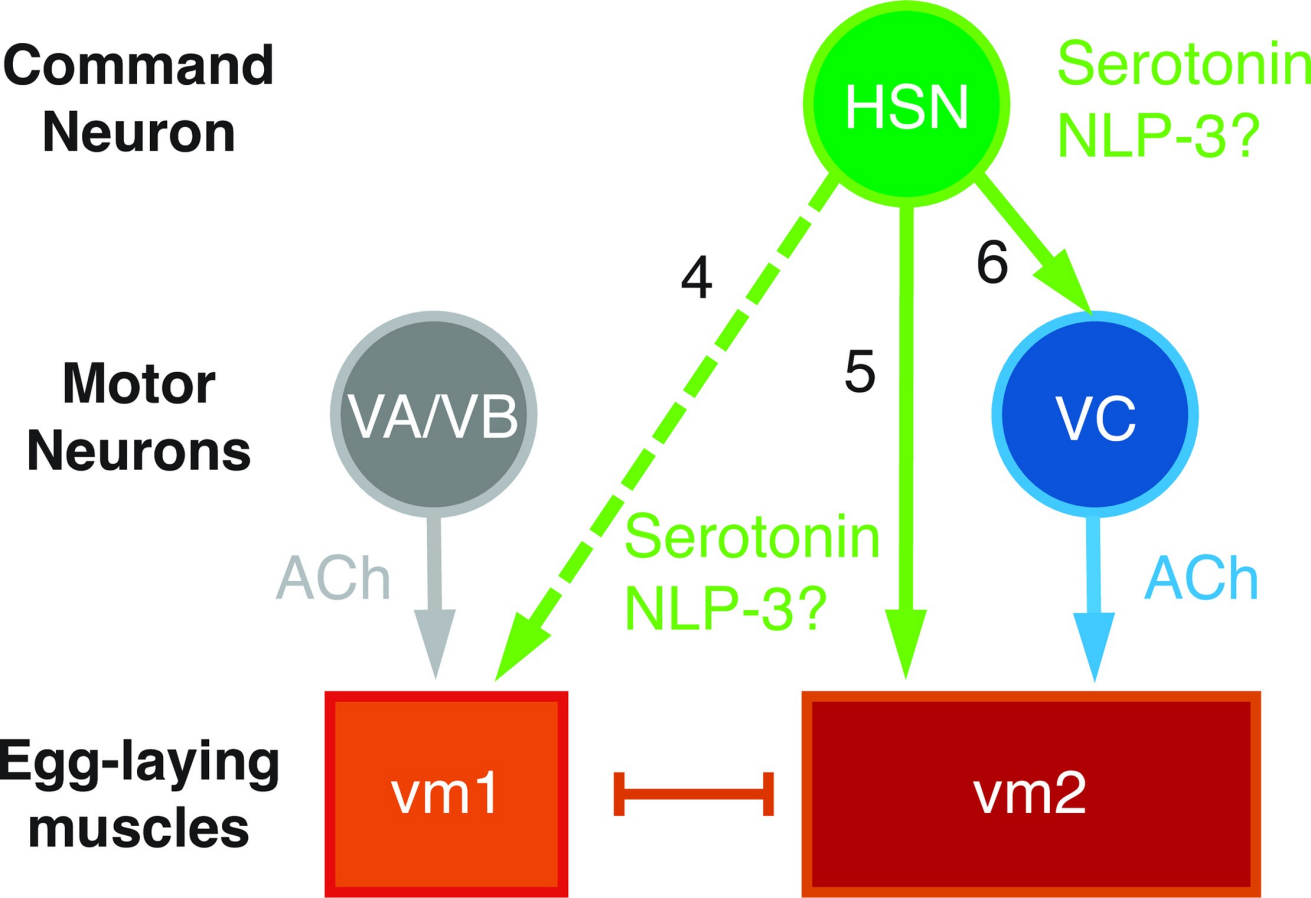
Model depicting signaling events that activate the egg-laying circuit. Solid arrows, synaptic signaling; dashed arrow, extrasynaptic signaling; and bar, gap junctions. vm1 and vm2 cells are known to express multiple serotonin receptor isoforms that each mediate activation of egg laying. The signaling of serotonin onto VC neurons (arrow 3) remains hypothetical since no serotonin receptors have yet been described as expressed on these neurons. The possible direct signaling of NLP-3 depicted onto vm1 (arrow 2), vm2 (arrow 2), or and/or VC (arrow 3) is also hypothetical: NLP-3 receptors have yet to be identified and it thus remains unknown which cell(s) of the circuit express them.

How do serotonin and NLP-3 peptides activate the vm2 muscle cells? The receptor(s) for NLP-3 have not yet been identified in the egg-laying circuit, and it thus remains to be determined if such a receptor is expressed on vm2 and furthermore whether NLP-3 acts directly on these muscle cells. Previous studies demonstrated that serotonin activates egg laying via three G protein coupled serotonin receptors, SER-1, SER-5, and SER-7 [49]. Promoter::GFP transgenes for all three receptors show expression in the vm cells [43,45,33,49,50], but the transgenic animals carrying these GFP reporters have not been examined carefully to determine which receptors are expressed in vm1 versus vm2 cells. Even so, the published images of these animals suggest that both vm1 and vm2 express one or more of these GPCRs. Thus, the anatomy of the egg-laying circuit and serotonin receptor expression patterns suggest that HSNs release serotonin at synapses onto vm2 cells to directly activate these muscles. We note, however, that serotonin and/or NLP-3 likely also activate vm2 via an indirect route. Our past Ca^2+^ imaging studies [25] show that the HSNs activate the cholinergic VC motor neurons, which in turn directly synapse onto vm2 (Fig 1). No serotonin receptors are known to be expressed on the VC neurons, so NLP-3 may be the HSN-released signal that activates the VCs. We therefore propose a model (Fig 9) in which the HSNs activate the egg-laying circuit via two mechanisms: 1) Serotonin released by HSNs directly onto vm2 would act via G protein-coupled serotonin receptors to increase the excitability of the vm2 cells; and 2) NLP-3 peptides released by the HSNs would activate the VC neurons to release acetylcholine directly onto vm2, triggering vm2 contractions, that when concurrent with vm1 contractions result in egg release. We note that the mechanisms in the above model remain hypothetical, and in particular it remains to be tested experimentally if NLP-3 acts on the VC neurons. A crucial step towards this would be to identify NLP-3 receptors, which is necessary to determine in what cells of the egg-laying circuit these receptors are expressed and function to stimulate egg laying.

A surprising finding of this work is that activity of the vm1 muscle cells has little or no dependence on the HSNs or the signals they release. The vm1 and vm2 muscle cells are connected by gap junctions [46] and it was previously assumed that vm1 and vm2 cells would be efficiently electrically coupled and contract as a unit in response to excitatory signals released by HSNs onto vm2. To the contrary, by adjusting our Ca^2+^ imaging conditions to spatially resolve vm1 and vm2 cells, we found that this assumption is incorrect. In wild-type animals, most vm Ca^2+^ transients occur in vm1 only, and these do not result in egg release. Only during the ~2 minute active state of the egg-laying circuit when the HSNs show rhythmic activity [25] did we observe vm1 + vm2 transients, during which all observed egg release events occurred. It may be that vm1-only transients result from release of acetylcholine by the VA7 and VB6 ventral cord motor neurons, which synapse directly onto the vm1s [23,47]. We note that the SER-1, SER-5, and SER-7 G protein-coupled serotonin receptors that promote egg laying appear to be expressed on both the vm1 and vm2 cells [43,45,33,49,50]. Thus while HSN serotonin released synaptically onto vm2 appears to act directly on vm2 to help excite these cells, it may also act extrasynaptically to promote vm1 response to both acetylcholine from VA/VB and to depolarization via gap junctions from vm2. In this way, serotonin would promote the simultaneous contraction of both vm1 and vm2 cells. HSN-released serotonin and/or NLP-3 also may help produce simultaneous contraction of the vm cells found on the anterior and posterior sides of the vulval opening. These two sets of vm cells are not physically connected [46] except that the HSNs form synapses onto the muscle arms extending from both the anterior and posterior vm2 cells (Fig 1). By simultaneously releasing their signals onto both the anterior and posterior vm2 muscle arms, the HSNs promote the coordinated contraction of both the anterior and posterior vm cells necessary for successful egg release [51].

### Co-release of small molecule neurotransmitters and neuropeptides is a widespread phenomenon

The apparent co-release of serotonin and NLP-3 from the HSN neurons is just one instance of the broad but poorly-studied phenomenon of co-transmission by small-molecule neurotransmitters and neuropeptides. Most neurons release both small-molecule neurotransmitters and neuropeptides. This issue has been analyzed in greatest detail within the *C*. *elegans* nervous system. There are seven known small-molecule neurotransmitters in *C*. *elegans*, and 107 of the 118 neuron types in *C*. *elegans* hermaphrodites express at least one of them [52]. At least 95 *C*. *elegans* neuropeptide genes have been described, including 23 FLP genes encoding FMRFamide-related peptides, 32 NLP genes encoding neuropeptide-like proteins, and 40 INS genes encoding insulin-like peptides. Promoter::GFP fusion transgenes have been generated for all 95 of these neuropeptide genes to analyze their expression patterns. The individual neurons expressing each FLP gene were identified, and >50% of *C*. *elegans* neurons express at least one FLP peptide gene [36]. Although the individual cells expressing each NLP and INS gene have not yet been identified, images of the expression patterns show that the large majority of these peptide genes are expressed complex subsets of neurons [35]. Thus, we can infer that the typical neuron in *C*. *elegans* releases one small-molecule neurotransmitter and one or more type of neuropeptide. Similarly, the presence of both small-molecule neurotransmitters and neuropeptides within the same individual neurons is widespread in both *Drosophila* [53,54] and in mammals [55].

The functional consequences of a neuron releasing two different types of signaling molecules have been difficult to study with precision in the complex circuits of the mammalian brain, but this issue has been the focus of many studies of small neural circuits in invertebrate model organisms [11,56]. In such small circuits, individual presynaptic neurons that co-release a small-molecule neurotransmitter and neuropeptides can be identified. The functional effects of each signal can be measured by bath application of neurotransmitter agonists/antagonists and/or neuropeptides followed by measurements of circuit activity using electrophysiological methods. Such work has led to a rich set of findings and many different schemes for the use of co-transmission within circuits [11,56]. However, the limitations of these studies include that bath application of signaling molecules does not always mimic the effects of their release from neurons [11]. The genetic approaches for analyzing co-transmission described in this work provides a useful complement to electrophysiological studies as they permit manipulation of endogenous signaling molecules with mutations and transgenes, recording of circuit activity using genetically-encoded calcium indicators, and manipulation of neural activity using optogenetics, all within intact, freely-behaving animals. We are aware of just one previous study that focused on co-transmission using this combination of genetic approaches [18]. In that pioneering study, an odor was shown to cause a *C*. *elegans* sensory neuron to co-release glutamate and a neuropeptide to act on different interneurons. The glutamate evokes a behavioral response to the odor via a complex and incompletely understood motor circuit. The neuropeptide acts via a G protein-coupled receptor to cause release a second neuropeptide back onto the original sensory neuron, limiting activity of the sensory neuron and the timescale of the behavioral response to the odor.

Our studies of co-transmission focus on the *C*. *elegans* egg-laying circuit because its anatomical simplicity holds the potential that all the cells and signaling events that control this circuit can be defined, something that has yet to be accomplished for any neural circuit. In this study, we discovered that serotonin and NLP-3 peptides released from the HSN command neurons have parallel and partially redundant effects to activate coordinated, rhythmic contraction of the egg-laying muscles. This finding may be analogous to results of some previous studies of co-transmission, in which the two co-released signals act convergently to increase activity the same target cells. The most relevant example is in the mammalian brain respiratory circuit, where co-release of serotonin and the neuropeptide Substance P have parallel effects promoting rhythmic circuit activity [10]. It will be interesting to determine how mechanistically analogous these two cases of serotonin/neuropeptide co-transmission actually are, and whether the action of serotonin within the *C*. *elegans* egg-laying circuit will provide a model for the detailed workings of serotonin within neural circuits of the human brain.

## Methods

### *C*. *elegans* strains

*C*. *elegans* strains were cultured at 20°C on NGM agar plates with *E*. *coli* strain OP50 as a food source [57]. All strains were derived from the Bristol N2 wild-type strain. Genetic crosses and generation of transgenic strains were by standard methods [58,59]. Table 1 shows a list of strains, mutants, and transgenes used in this study.

**Table 1:** Strains used in this study.

Strain	Feature	Genotype	Figures
**N2**	Bristol strain	Wild type	1,2
**MT2059**	Lacks HSN neurons	*egl-1(n986dm)* V	1,2
**MT15434**	Lacks serotonin	*tph-1(mg280)* II	1,2
**LX1836**	*egl-6*::ChR2::YFP	*wzIs30 IV; lite-1(ce314) lin-15(n765ts) X*	1,3
**MT8189**	Strain for transgene production	*lin-15(n765ts) X*	2
**LX1954**	*nlp-3* overexpressor	*lin-15(n765ts)* X; *vsEx748*	2
**LX1955**	*nlp-8* overexpressor	*lin-15(n765ts)* X; *vsEx749*	2
**LX1956**	*nlp-15* overexpressor	*lin-15(n765ts)* X; *vsEx750*	2
**LX1957**	*flp-5* overexpressor	*lin-15(n765ts)* X; *vsEx751*	2
**LX1981**	*flp-19* overexpressor	*lin-15(n765ts)* X; *vsEx757*	2
**LX1978**	*nlp-3* null mutant	*nlp-3(tm3023)* X	2,6
**LX2366**	double mutant	*tph-1(mg280)* II; *nlp-3(tm3023)* X	2
**MT15951**	*nlp-3* null mutant	*nlp-3(n4897)* X	2
**LX2388**	outcrossed MT15951 8x by mating to N2	*nlp-3(n4897)* X	2
**LX2389**	double mutant	*tph-1(mg280)* II; *nlp-3(n4897)* X	2
**LX2090**	vm mCherry, mated with animals carrying a pJB11 *nlp-3*::GFP transgene	*lin-15(n765ts)* X; *vsEx780*	3A
**LX1836**		*wzIs30* IV; *lite-1(ce314) lin-15(n765ts)* X	3C
**LX1832**	mated to LX1836 for Fig 3C “control”	*lite-1(ce314) lin-15(n765ts)* X	3C
**LX1837**		*tph-1(mg280)* II; *wzIs30* IV; *lite-1(ce314) lin-15(n765ts)* X	3C
**LX2335**	*mated to LX1837 for Fig 3C “tph-1”*	*tph-1(mg280)* II; *lite-1(ce314) lin-15(n765ts)* X	3C
***LX2367***		*wzIs30* IV; *lite-1(ce314) nlp-3(tm3023) lin-15(n765ts)* X	3C
**LX2364**	mated to LX2367 for Fig 3C *“nlp-3”*	*lite-1(ce314) nlp-3(tm3023) lin-15(n765ts)* X	3C
**LX2368**		*tph-1(mg280)* II; *wzIs30* IV; *lite-1(ce314) nlp-3(tm3023) lin-15(n765ts)* X	3
**LX2365**	mate to LX2368 for Fig 3C *“tph-1; nlp-3”*	*tph-1(mg280)* II; *lite-1(ce314) nlp-3(tm3023) lin-15(n765ts)* X	3
**LX2526**	*rab-*3::NLS::tagRFP and *nlp-3*::GFP	*otIs356* V; *lin-15(n765ts)* X; *vsEx946*	4, S3, S4 vid
**LX2518**	*nlp-3* overexpressor	*vsIs276* II	5
**LX2519**	*nlp-3* overexpressor	*vsIs275* III	
***LX2521***	*nlp-3* overexpressor lacking HSNs	*vsIs275* III; *egl-1(n986dm)* V	5
**LX2522**	*nlp-3* overexpressor lacking HSNs	*vsIs276* III; *egl-1(n986dm)* V	5
**DA1814**	Serotonin receptor 1 deletion	*ser-1(ok345)* X	6
**LX2392**	“control” in Fig 6B	*lin-15(nt65ts)* X; *vsEx885*	6
**LX2394**	“*nlp-3* ox” in Fig 6B	*lin-15(nt65ts)* X; *vsEx887*	6
**LX2393**	“control in tph-1” in Fig 6B	*tph-1(mg280)* II; *lin-15(nt65ts)* X; *vsEx886*	6
**LX2395**	“*nlp-3* ox in *tph-1*” in Fig 6B	*tph-1(mg280)* II; *lin-15(nt65ts)* X; *vsEx888*	6
**LX1918**	“wild type” in Figs 7 and 8	*vsIs164 lite-1(ce314) lin-15(n765ts)* X	7/8
**LX1937**	“*tph-1*” in Figs 7 and 8	*tph-1(mg280)* II; *vsIs164 lite-1(ce314) lin-15(n765ts)* X	7/8
**LX2369**	“*nlp-3*” in Figs 7 and 8	*vsIs164 lite-1(ce314) nlp-3(tm3023) lin-15(n765ts)* X	7/8
**LX2370**	“*tph-1; nlp-3*” in Figs 7 and 8	*tph-1(mg280)* II; *vsIs164 lite-1(ce314) nlp-3(tm3023) lin-15(n765ts)* X	7/8
**LX1938**	“*egl-1*” in Figs 7 and 8	*egl-1(n986dm)* V*; vsIs164 lite-1(ce314) lin-15(n765ts)* X	7/8
**LX2527**	*nlp-3*::GFP and *eat-4*::NLS::mCherry	*him-5(e1490)* V; *otIs518*; *vsEx948*	S3
**LX2529**	*nlp-3*::GFP and *cho-1*::NLS::mCherry	*him-5(e1490)* V; *otIs544*; *vsEx950*	S3
**LX2523**	*nlp-3*::GFP, used for dye filling	*lin-15(n765ts)* X; *vsEx944*	S3

Gene deletion strains for *nlp-3* [60], *tph-1* [61], and *ser-1* [43] were outcrossed four to ten times to the wild-type strain. The *nlp-3*(*tm3023*) allele is a 354 bp deletion that removes DNA flanked by the sequences GTCTGGACGGAAAGATCGTT…CGTGAGACTAGAAGTCCAC. The *nlp-3*(*n4897*) allele is a 1405 bp deletion that removes DNA flanked by the sequences TCCCGGATTAGTGTCCAGTC…TATGTTCAACCGAAATTAAA. Each gene deletion used removes a portion or all of the promoter and/or coding sequences of the corresponding gene such that no functional gene product is expected. The genotypes for all strains constructed using these deletions were verified by agarose gel analysis of PCR amplification products from the corresponding genes.

### Egg-laying behavioral assays

Quantitation of unlaid eggs in adult animals and percentage of early-stage eggs laid was performed using adult animals 30 hours after staging as late L4 larvae as described in [39].

### Optogenetic assays

HSN neurons were optogenetically activated in animals carrying the *wzIs30* transgene, which expresses a Channelrhodopsin-2::yellow fluorescent protein (ChR2::YFP) fusion in the HSN and a few other neurons unrelated to the egg-laying circuit from the *egl-6a* promoter [62,63]. *wzIs30* also carries a *lin-15* marker plasmid that rescues the multivulva phenotype of *lin-15(n765ts)* mutant animals. All animals used in optogenetic assays were homozygous for the *lite-1(ce314)* mutation to eliminate an endogenous avoidance response of *C*. *elegans* to blue light. The *wzIs30* transgene was homozygous for the experiment shown in Fig 1E, but we noticed that the homozygous transgene caused developmental defects in the HSNs of some animals (S1 Fig) that resulted in these animals being egg-laying defective. Therefore, for the experiment in Fig 1E, we examined the animals prior to optogenetic stimulation and discarded the small percentage of animals that were visibly egg-laying defective. The experiment shown in Fig 3C was carried out such that all animals were *wzIs30*/+ heterozygotes, which we found had morphologically normal HSNs (S1 Fig). First, we constructed the strains indicated in Table 1 that were homozygous for *wzIs30* and also homozygous for the other mutations required by the experiment. Then, we generated males of each of these strains and mated them to corresponding strains that were genetically identical except that they lacked *wzIs30*. The cross progeny, identified by the presence of YFP-labeling, thus were heterozygous for *wzIs30* but homozygous for all other mutations used in the experiment.

ChR2 expressing strains were grown in the presence or absence of the ChR2 cofactor all-trans retinal (ATR). ATR was prepared at 100 mM in 100% ethanol and stored at -20° C. To prepare NGM plates for behavior analysis, ATR was diluted to 0.4 mM with room temperature cultures of OP50 bacteria in B Broth, and 200 μl of culture was seeded onto each 60 mm NGM plate. The plates were allowed to grow for 24 hr at 25–37°C, after which late L4 worms were staged onto prepared plates for behavioral assays 24 hr later. To begin an assay, a video recording was initiated (Flea 3, 0.3 Megapixel, FireWire CCD camera, Point Grey Research) and simultaneously a shutter was opened on a EL6000 metal halide light source generating 3.2 mW/cm2 of 470 ± 20 nm blue light via a EGFP filter set mounted on a Leica M165FC stereomicroscope.

### Molecular biology and transgenes

To overexpress neuropeptide genes (Fig 2A), fosmid genomic clones including individual neuropeptide genes were selected from the *C*. *elegans* fosmid library (Source BioScience). The fosmids used for four of the neuropeptide genes were: *nlp-3*, WRM0633dC06; *nlp-8*, WRM0614aB10; *nlp-15*, WRM066cH12; *flp-5*, WRM0622aF03. For overexpression of a fifth neuropeptide gene, *flp-19*, we instead PCR amplified genomic DNA containing the 746 bp *flp-19* coding region along with 5015 bp upstream and 1155 bp downstream (primers used were 5’- tcttaccaatattccggttagtgtcc-3’ and 5’-gtaatgtaagaaataattcgagccacg-3’). Multicopy extrachromosomal transgenes were generated for each neuropeptide gene by microinjection, using the fosmid or PCR product at 50 ng/μl along with the *lin-15* rescuing plasmid pL15EK at 50 ng/μl into *lin-15(n765ts*) mutant animals. Negative controls were injected with pL15EK without any neuropeptide gene. Five independent transgenic overexpressor lines were generated for each injection and Fig 2A shows data averaged from these. Table 1 lists one representative overexpressor strain for each neuropeptide gene.

To determine the effects of overexpressing *nlp-3* in animals lacking serotonin (Fig 6B), either a ~5 kb PCR product containing the *nlp-3* gene (primers used were 5'-accaagctaatcaaattttgtcaccg-3' and 5'-gcaatacaaccaatcccttttcatctc-3’) or as a control, *E*. *coli* genomic DNA digested to an average size of ~5 kb, was injected at 10 ng/μl along with 50 ng/μl of the *lin-15* rescuing plasmid pL15EK into either *lin-15* or *tph-1; lin-15* animals, and transgenic lines were identified by rescue of the *lin-15* phenotype. Five independent transgenic lines were established for each injection, and the early stage egg assay [39] was carried out on 50 eggs per line (250 eggs total per condition tested). One representative line for each condition is listed in Table 1.

To determine the effects of overexrpessing *nlp-3* in animals lacking HSN neurons, we generated chromosomally-integrated *nlp-3* overexpressing transgenes. Primers 5' cagtcagtcgacgcaatacaaccaatcccttttcatctc 3' and 5' cagtcaggtaccaccaagctaatcaaattttgtcaccg 3' were used to amplify the *nlp-3* gene from fosmid genomic clone wrm0613cB03, generating a PCR product with ~3700 bp of promoter and ~900 bp of 3' untranslated region. This was digested with restriction enzymes SalI and KpnI and inserted into Sal1 and Kpn1 digested plasmid vector pUC19. The resulting clone pAO30 was microinjected into *C*. *elegans* at 60 ng/μl along with 10 ng/μl of pCFJ90 (a *myo-2*::mCherry marker plasmid) and 25 ng/μl of genomic DNA from E. coli strain DH5α digested to an average size of 5 kb. The resulting transgene was chromosomally integrated after psoralen/UV mutagenesis to produce the integrated transgenes *vsIs275* III and *vsIs276* II.

To visualize *nlp-3* expression, we made the plasmid pJB11, a derivative of pJB9 [25], which in turn is a derivative of pPD49.26 (Fire lab *C*. *elegans* vector kit). pJB11 has a ~4 kb *nlp-3* promoter region inserted into multiple cloning site I (MCS I) of pPD49.26, followed by the GFP coding sequence inserted into MCSII, and by ~750 bp of the *nlp-3* 3' untranslated region inserted into MCSIII.

### Identification of *nlp-3*::GFP expressing cells

The *nlp-3*::GFP plasmid pJB11 was transformed into *C*. *elegans* by microinjection at 20 ng/μl along with 25 ng/μl of genomic DNA from *E*. *coli* strain DH5α digested to an average size of 5 kb and 50 ng/μl of the *lin-15* rescuing plasmid pL15EK into strains carrying the *lin-15(n765ts)* marker mutation. The strains injected carried marker transgenes that label specific subsets of neurons with red fluorescent proteins. These transgenes were *otIs356*, which expresses nuclear-localized tagRFP in all neuronal nuclei from the *rab-3* promoter [64], *otIs518*, which expresses nuclear-localized mCherry in glutamatergic neurons [65], and *otIs544*, which expresses nuclear-localized mCherry in cholinergic neurons [66]. The *nlp-3*::GFP transgene was also transformed into a strain not expressing any red fluorescent protein, and the resulting animals were stained with DiD, a red fluorescent dye which labels a specific subset of sensory neurons [67]. The double-labeled strains were imaged using an LSM880 confocal microscope, and the GFP labeled cells were identified based on position, morphology, and comparison to the known identities of the red-labeled neurons [68]. S3 Fig shows representative head images of animals expressing *nlp-3*::GFP and showing labeling with each of the red fluorescent markers used.

### Ratiometric calcium imaging

Freely-behaving animals were mounted between a glass coverslip and a chunked section of an NGM plate for imaging as described [23,47,48]. Two channels were recorded with a 20X Plan-Apochromat objective (0.8 NA) using a Zeiss LSM 710 Duo LIVE head. Recordings were collected at 20 fps at 256 x 256 pixel, 16 bit resolution, for 1 hour. Five 1 hour recordings were collected for each genotype studied, and Figs 7 and 8 present analysis of the data from three representative 1 hour recordings per genotype. The stage and focus were adjusted manually to keep the egg-laying system in view and focused during recording periods. Care was taken to find a lateral focus that included as much of the vm1s and vm2s as possible. Ratiometric analysis for Ca^2+^ recordings was performed in Volocity (version 6.3, PerkinElmer). Volocity was also used to identify the vulval muscles as the region of interest (ROI) analyzed in each video frame using size and intensity parameters that varied over a small range based on individual animals. Any misidentified objects were manually excluded prior to final analysis. A ratio channel was calculated from GCaMP5 (GFP) and mCherry fluorescence channels within the ROI. The lowest 10% of the GCaMP5/mCherry ratio values within a 1 hour recording were averaged to establish a Δ*R*/*R* baseline using a custom MATLAB script. This script also identifies the peak of a transient based on identifying a change in prominence that was typically 0.25 ΔR/R over the preceding second, but this was adjusted based on the smoothness of the data for individual animals. With the experimenter blinded to the genotype of the animals being scored, video of each peak was observed in the ratio channel to determine whether the indicated activity was restricted to vm1 or present in both vm1 and vm2 and whether an egg was laid. We scored a transient as vm1-only if it was clear in the ratio channel that there was a difference of more than 50% of maximum activity between the vm1s and the adjacent regions where vm2 cells were located.

### Statistical methods

Statistical analyses were performed using GraphPad Prism for Mac OS X v. 7.0a. 95% confidence intervals were determined and 1- or 2-way ANOVA, corrected for multiple comparisons, were performed to determine statistical significance. For egg stage assays, we used the Wilson-Brown method for determining the 95% confidence intervals for binomial data and used a Bonferroni correction to correct for multiple comparisons.

## Supporting information

S1 Fig Animals homozygous for the *egl-6*::ChR2-YFP transgene have visibly defective HSNs. **A-B)** Vulval region of an adult homozygote for the *egl-6p*::ChR2::YFP transgene. Abnormal HSN morphology can be seen by comparing to normal HSN morphology in S1B Fig. Asterisk, location of the vulva; filled arrowhead, HSN cell body; open arrowhead, HSN presynaptic varicosity. The morphology of the HSN in the homozygote is too abnormal to confidently identify a cell body or synapse. **B)** Vulval region of an adult heterozygote for the *egl-6p*::ChR2::YFP transgene showing a wild-type HSN morphology. This is the same image seen in Fig 3B, repeated here for comparison to S1A Fig.
(PDF)

S2 Fig A *tph-1* null mutation does not detectably affect egg laying upon optogenetic activation of HSNs. Measurements of egg laying upon blue light stimulation in *egl-6p*::ChR2-YFP/+ animals that were controls (wild type for *tph-1* and *nlp-3*) or that carried null mutations in *tph-1* or *nlp-3*. All animals also were homozygous for a *lite-1(ce314)* mutation that eliminates endogenous responses to blue light. **A)** Average time from onset of blue light simulation to first egg laid, measured as in [63]. There was no significant difference between control and *tph-1* animals, but *nlp-3* animals initiated egg laying more slowly and with higher animal-to-animal variability, and 7/20 *nlp-3* animals tested failed to lay any eggs. Center line is the mean, error bars are 95% confidence intervals. n.s., no significant difference, *, p<0.033, ***, p<0.001. **B)** Cumulative distribution plot of the time to last egg laid during the 60 second blue light illumination experiment. One Gaussian curve fit both control and *tph-1* data for last egg laid during the assay. **C)** Plots of raw data showing the time point of each egg laid by each genotype. Each of 20 animals tested per genotype is represented by a vertical column, with each point indicating the time after the onset of blue light illumination when an individual egg was laid. Empty columns indicate that no eggs were laid. Two or three horizontally adjacent points indicate eggs laid simultaneously within the 0.05 sec time resolution of our video recording.
(PDF)

S3 Fig Head images of animals expressing *nlp-3*::GFP with four different red fluorescent markers that label different identified subsets of neurons. Three markers are transgenes that express red fluorescent proteins from promoters expressed in **A)** ~all neurons, *rab-3* promoter; **B)** glutamatergic neuron, *eat-4* promoter; or **C)** cholinergic neuron, *cho-1* promoter. **D)** shows an *nlp-3*::GFP animal stained with the red fluorescent dye DiD, which labels a subset of sensory neurons. All images are shown with anterior to the left, ventral down, and are two-dimensional representations prepared from three-dimensional confocal images that have been cropped in the Z dimension so that only the left side of the head is shown. Not all cells that express *nlp-3*::GFP or the red fluorescent labels are visible at the display brightness levels chosen for these images. Images shown are representative of those used to make *nlp-3*::GFP expressing cell identifications. Cell identifications were based on three-dimensional analysis of multiple images of each body region with each of the red markers.
(PDF)

S1 Table Raw data used to generate graphs in this work. Raw numerical data used to generate each graph presented in this work is shown in tabular form.
(DOCX)

S1 Video Optogenetic activation of HSNs in both wild-type and *tph-1* mutant animals results in egg laying. Videos showing similar egg-laying responses upon optogenetic activation of HSNs in one animal wild-type for *tph-1* ("wild type") or carrying a *tph-1* null allele ("no serotonin"). Both animals shown carry the same transgene expressing ChR2 in the HSNs. Fluorescent powder is sprinkled on the Petri dishes so that the onset of blue light activation of ChR2 can be seen (at 9 seconds into the video for the wild type, 29 second for the *tph-*1 mutant). Laid eggs are outlined in green to make them easier to discern.
(MP4)

S2 Video Methods used to measure Ca^2+^ transients in vm1/vm2 vulval muscle cells of freely-behaving animals. Recordings at 20 frames per second were made of vulval muscle Ca^2+^ transients in freely-moving animals by continuously moving the microscope stage to keep the egg-laying system centered in the field of view. One-hour recordings were made of each animal. This video is a short clip of one video recording including two egg-laying events. Animal used in these recordings co-expressed in the vm1 and vm2 vulval muscles both the Ca^2+^-insensitive red fluorescent protein mCherry (left panel in video) and the Ca^2+^-sensitive green fluorescent protein GCaMP5 (right panel). The center panel shows a computed false-colored image of the ratio of GCaMP5/mCherry fluorescence, with higher Ca^2+^ levels indicated by colors more toward the red end of the color spectrum. This false-color image is superimposed on a bright-field image of the worm, which allows egg release events to be observed. Video analysis software was used to analyze the mCherry channel to automatically detect the region of the vm cells in each video frame, and to then compute the mean GCaMP5 fluorescence intensity within this region, as graphed at bottom of the center panel. The clip shown includes a low intensity Ca^2+^ transient (15 seconds into the video) and two high-intensity Ca^2+^ transients that are each accompanied by release of an egg (at 18 and 22 seconds into the video). The medial focus used in this video makes it more difficult to completely resolve vm1 and vm2 cells than the more lateral focus shown in S3 Video.
(MP4)

S3 Video Lateral focus allows vm1-only Ca^2+^ transients to be resolved. Video clip of a Ca^2+^ recording illustrating how a more lateral focus allows vm1 and vm2 cells to be resolved so that Ca^2+^ transients can be observed to sometimes occur in vm1 cells only. As in S2 Video, the image uses false-coloring to indicate the ratio of GCaMP to mCherry fluorescence, with colors at the blue end of the spectrum indicating low Ca^2+^, and colors at the yellow/red end of the spectrum indicating higher Ca^2+^. The initial segment of the video is focused more laterally (as in the S2 Video), which does not allow individual vm1 and vm2 cells to be identified. The video then shifts to a more lateral focus that does all individual muscle cells to be discerned. At the end, the video freezes during a Ca^2+^ transient that can be seen to occur in just a single vm1 cell.
(MP4)

S4 Video Three dimensional rotation of the head of a transgenic worms expressing *nlp-3*::GFP. This animal also expresses a *rab-3*::NLS::tagRFP transgene that marks ~all neuronal nuclei with red fluorescence. Partway through the video, colored spheres marking each GFP-expressing cell body are made visible with the identification of each marked cell indicated. "L" or "R" at the end of a cell designation indicates that the cell is the left or right member, respectively, of a bilaterally symmetric neuron pair. The AINL/R cells are not visibly GFP labeled in this particular animal. The image displayed is one of >40 three-dimensional head images examined to make the cell identifications shown, and the *rab-3* marker shown is one of four red fluorescent markers used.
(MP4)
