# Supplementary figures and images for: Serotonin and neuropeptides are both released by the HSN command neuron to initiate Caenorhabditis elegans egg laying
Source: PLoS Genet. 2019 Jan 24;15(1):e1007896. doi: 10.1371/journal.pgen.1007896 (PMC6363226; doi:10.1371/journal.pgen.1007896)

Figure S1

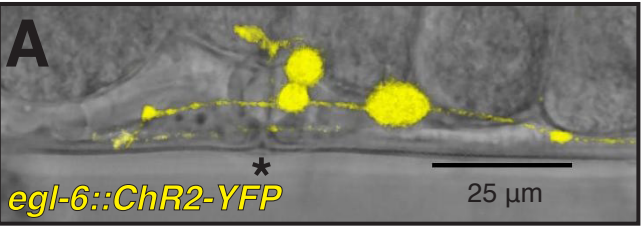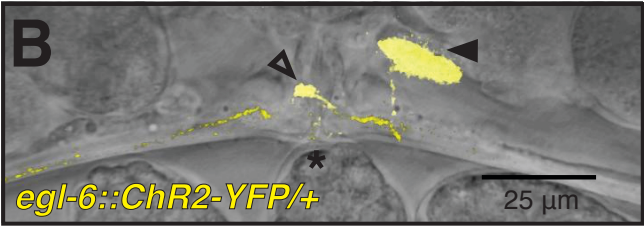

Supplement: S1 Fig — A-B) Vulval region of an adult homozygote for the egl-6p::ChR2::YFP transgene. Abnormal HSN morphology can be seen by comparing to normal HSN morphology in S1B Fig. Asterisk, location of the vulva; filled arrowhead, HSN cell body; open arrowhead, HSN presynaptic varicosity. The morphology of the HSN in the homozygote is too abnormal to confidently identify a cell body or synapse. B) Vulval region of an adult heterozygote for the egl-6p::ChR2::YFP transgene showing a wild-type HSN morphology. This is the same image seen in Fig 3B, repeated here for comparison to S1A Fig. (PDF) [file pgen.1007896.s001.pdf]

Figure S2

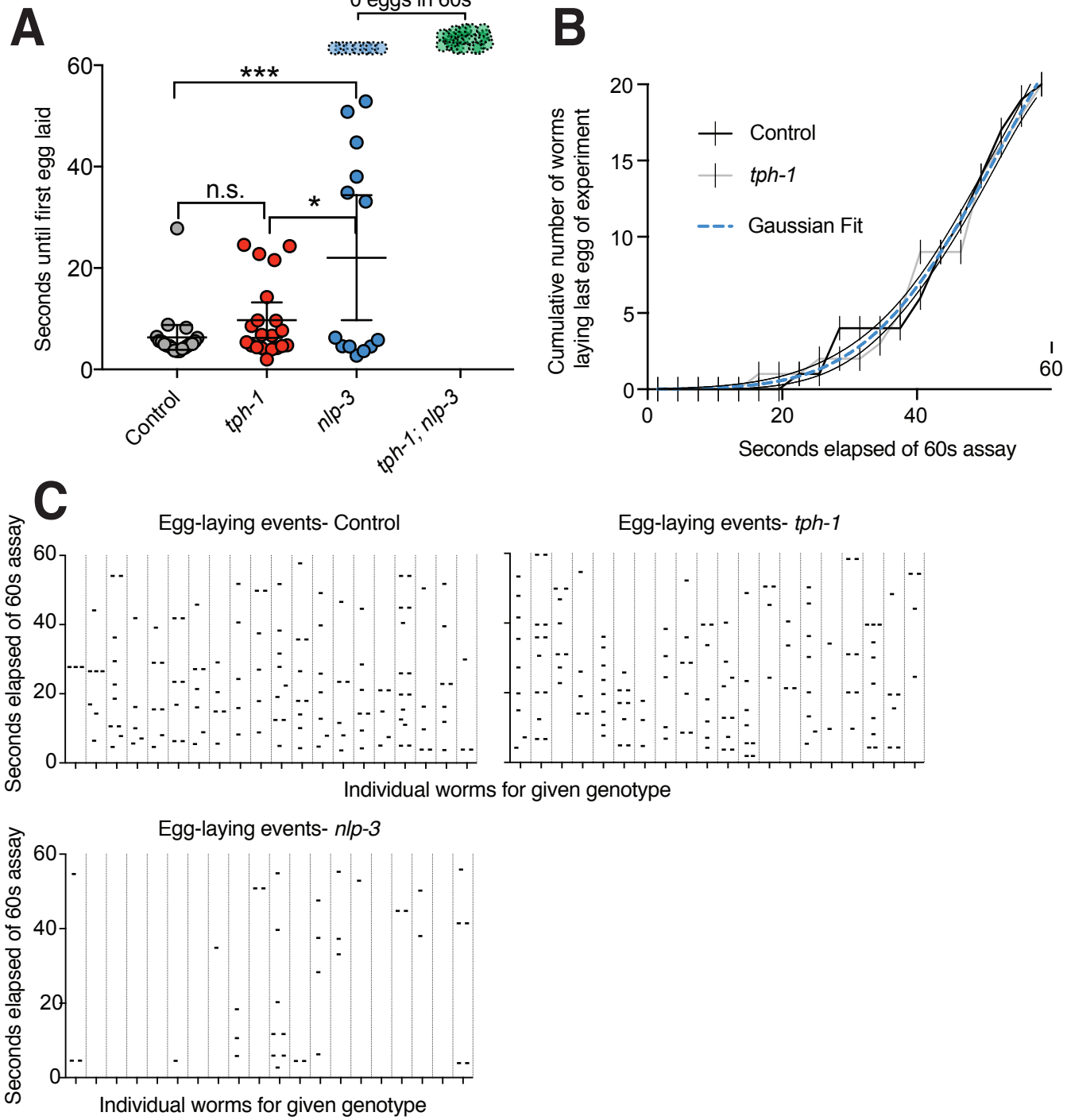

Supplement: S2 Fig — Measurements of egg laying upon blue light stimulation in egl-6p::ChR2-YFP/+ animals that were controls (wild type for tph-1 and nlp-3) or that carried null mutations in tph-1 or nlp-3. All animals also were homozygous for a lite-1(ce314) mutation that eliminates endogenous responses to blue light. A) Average time from onset of blue light simulation to first egg laid, measured as in [63]. There was no significant difference between control and tph-1 animals, but nlp-3 animals initiated egg laying more slowly and with higher animal-to-animal variability, and 7/20 nlp-3 animals tested failed to lay any eggs. Center line is the mean, error bars are 95% confidence intervals. n.s., no significant difference, *, p<0.033, ***, p<0.001. B) Cumulative distribution plot of the time to last egg laid during the 60 second blue light illumination experiment. One Gaussian curve fit both control and tph-1 data for last egg laid during the assay. C) Plots of raw data showing the time point of each egg laid by each genotype. Each of 20 animals tested per genotype is represented by a vertical column, with each point indicating the time after the onset of blue light illumination when an individual egg was laid. Empty columns indicate that no eggs were laid. Two or three horizontally adjacent points indicate eggs laid simultaneously within the 0.05 sec time resolution of our video recording. (PDF) [file pgen.1007896.s002.pdf]

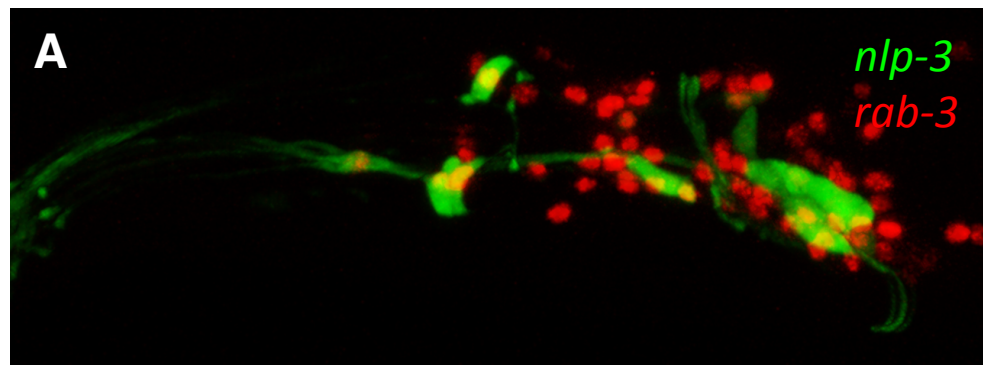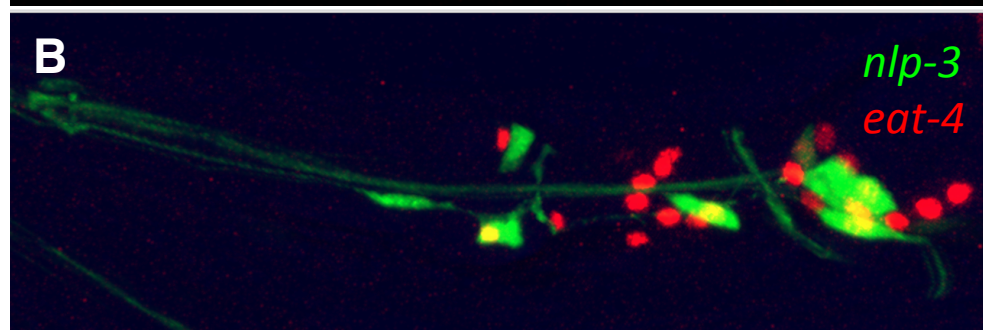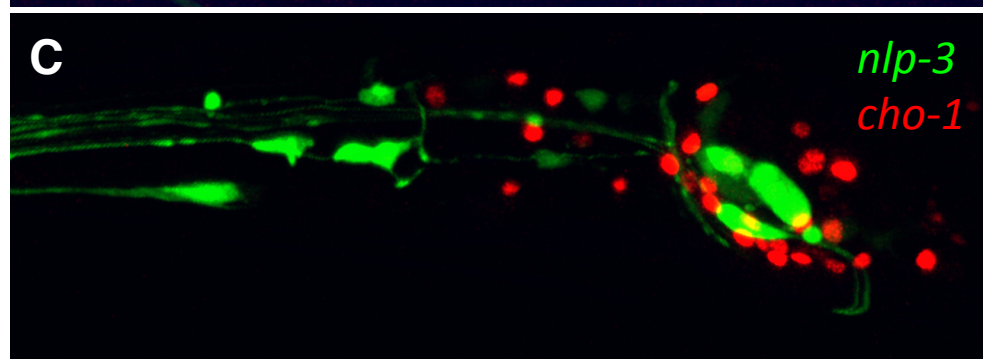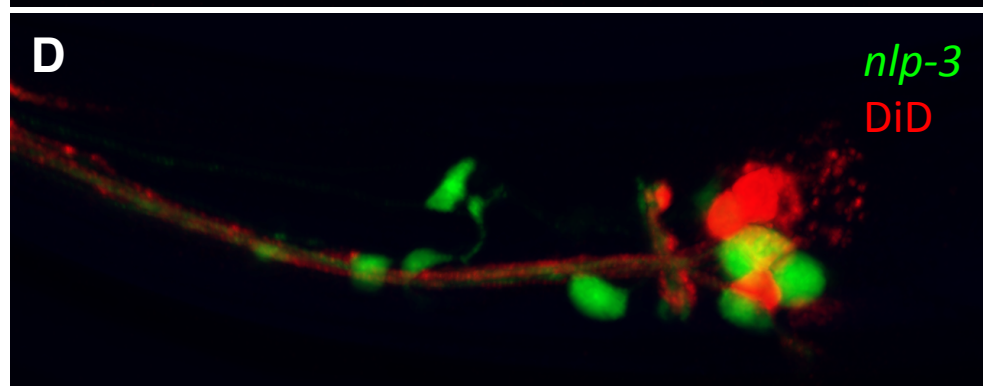

**S3 Fig**

Supplement: S3 Fig — Three markers are transgenes that express red fluorescent proteins from promoters expressed in A) ~all neurons, rab-3 promoter; B) glutamatergic neuron, eat-4 promoter; or C) cholinergic neuron, cho-1 promoter. D) shows an nlp-3::GFP animal stained with the red fluorescent dye DiD, which labels a subset of sensory neurons. All images are shown with anterior to the left, ventral down, and are two-dimensional representations prepared from three-dimensional confocal images that have been cropped in the Z dimension so that only the left side of the head is shown. Not all cells that express nlp-3::GFP or the red fluorescent labels are visible at the display brightness levels chosen for these images. Images shown are representative of those used to make nlp-3::GFP expressing cell identifications. Cell identifications were based on three-dimensional analysis of multiple images of each body region with each of the red markers. (PDF) [file pgen.1007896.s003.pdf]
