# Supplementary material for: Serotonin and neuropeptides are both released by the HSN command neuron to initiate Caenorhabditis elegans egg laying
Source: PLoS Genet. 2019 Jan 24;15(1):e1007896. doi: 10.1371/journal.pgen.1007896 (PMC6363226; doi:10.1371/journal.pgen.1007896)
Supplement: S1 Table — Raw numerical data used to generate each graph presented in this work is shown in tabular form. (DOCX) [file pgen.1007896.s004.docx]

**S1 Table: Raw data used to generate graphs in this work.**

**Data Table 1: Figure 1E**

| + cofactor | |  | - cofactor | |
| --- | --- | --- | --- | --- |
| WT | *tph-1* |  | WT | *tph-1* |
| 4 | 8 |  | 0 | 0 |
| 6 | 6 |  | 2 | 0 |
| 6 | 9 |  | 0 | 0 |
| 9 | 8 |  | 0 | 0 |
| 10 | 6 |  | 0 | 0 |
| 3 | 10 |  | 0 | 0 |
| 3 | 8 |  | 0 | 0 |
| 1 | 2 |  | 0 | 0 |
| 8 | 6 |  | 0 | 0 |
| 8 | 8 |  | 0 | 0 |
| 6 | 4 |  | 0 | 0 |
| 5 | 6 |  | 1 | 0 |
| 10 | 6 |  | 0 | 0 |
| 6 | 5 |  | 1 | 0 |
| 4 | 5 |  | 0 | 0 |
| 3 | 5 |  | 0 | 0 |
| 6 | 7 |  | 0 | 0 |
| 4 | 4 |  | 0 | 0 |
| 6 | 4 |  | 0 | 0 |
| 4 | 4 |  | 0 | 0 |

**Data Table 2: Figures 1B-D; Figures 2B-D**

| wild type | *tph-1* | *egl-1* | *nlp-3 (tm3023)* | *tph-1; nlp-3 (tm3023)* | *nlp-3 (n4897)* | *tph-1; nlp-3 (n4897)* |
| --- | --- | --- | --- | --- | --- | --- |
| 15 | 24 | 47 | 22 | 42 | 18 | 53 |
| 15 | 19 | 38 | 14 | 42 | 21 | 60 |
| 8 | 22 | 57 | 19 | 43 | 21 | 50 |
| 13 | 17 | 45 | 22 | 36 | 15 | 44 |
| 7 | 14 | 46 | 16 | 41 | 26 | 45 |
| 8 | 8 | 46 | 20 | 43 | 19 | 48 |
| 17 | 17 | 40 | 14 | 39 | 18 | 54 |
| 14 | 15 | 41 | 17 | 42 | 22 | 48 |
| 10 | 11 | 44 | 15 | 46 | 23 | 51 |
| 14 | 27 | 56 | 21 | 43 | 16 | 47 |
| 14 | 18 | 48 | 19 | 44 | 18 | 46 |
| 13 | 21 | 45 | 25 | 43 | 24 | 38 |
| 7 | 20 | 37 | 14 | 41 | 21 | 52 |
| 13 | 16 | 53 | 15 | 39 | 25 | 40 |
| 15 | 10 | 47 | 15 | 37 | 21 | 44 |
| 16 | 17 | 47 | 19 | 45 | 22 | 46 |
| 16 | 19 | 43 | 21 | 36 | 20 | 42 |
| 15 | 20 | 50 | 18 | 44 | 26 | 51 |
| 13 | 17 | 39 | 32 | 42 | 23 | 58 |
| 8 | 18 | 45 | 15 | 41 | 25 | 43 |
| 13 | 19 | 56 | 24 | 51 | 18 | 46 |
| 11 | 24 | 51 | 16 | 42 | 16 | 45 |
| 16 | 21 | 47 | 26 | 40 | 23 | 39 |
| 9 | 23 | 54 | 22 | 55 | 18 | 42 |
| 10 | 16 | 42 | 14 | 37 | 19 | 40 |
| 8 | 15 | 36 | 15 | 39 | 16 | 35 |
| 11 | 15 | 50 | 23 | 34 | 24 | 43 |
| 12 | 22 | 52 | 20 | 47 | 25 | 46 |
| 14 | 16 | 48 | 15 | 44 | 19 | 57 |
| 16 | 20 | 46 | 23 | 36 | 17 | 44 |
| 14 | 19 |  |  |  |  |  |
| 12 | 15 |  |  |  |  |  |
| 16 | 18 |  |  |  |  |  |
| 12 | 23 |  |  |  |  |  |
| 13 | 26 |  |  |  |  |  |
| 17 | 25 |  |  |  |  |  |
| 13 | 19 |  |  |  |  |  |
| 14 | 22 |  |  |  |  |  |
| 14 | 18 |  |  |  |  |  |
| 16 | 16 |  |  |  |  |  |
| 11 | 20 |  |  |  |  |  |
| 14 | 15 |  |  |  |  |  |
| 14 | 18 |  |  |  |  |  |
| 9 | 15 |  |  |  |  |  |
| 12 | 17 |  |  |  |  |  |
| 10 | 17 |  |  |  |  |  |
| 13 | 23 |  |  |  |  |  |
| 13 | 20 |  |  |  |  |  |
| 13 | 18 |  |  |  |  |  |
| 12 | 22 |  |  |  |  |  |
| 14 | 19 |  |  |  |  |  |
| 14 | 17 |  |  |  |  |  |
| 13 | 20 |  |  |  |  |  |
| 15 | 19 |  |  |  |  |  |
| 15 | 20 |  |  |  |  |  |
| 13 | 25 |  |  |  |  |  |
| 13 | 23 |  |  |  |  |  |
| 15 | 20 |  |  |  |  |  |
| 11 | 16 |  |  |  |  |  |
| 16 | 18 |  |  |  |  |  |

**Data Table 3: Figure 2A**

|  | **Control** | | ***nlp-8*** | | ***flp-5*** | | ***nlp-3*** | | ***nlp-15*** | | ***flp-19*** | |
| --- | --- | --- | --- | --- | --- | --- | --- | --- | --- | --- | --- | --- |
| **Number of early stage eggs (out of 200)** | 8 | | 10 | | 8 | | 180 | | 1 | | 6 | |
|  |  | | | | | | | | | | | |
| **% Early Stage Eggs** | 4.0 | | 5.0 | | 4.0 | | 90.0 | | 0.50 | | 3.0 | |
|  |  | | | | | | | | | | | |
| **95% Confidence Intervals** | + | - | + | - | + | - | + | - | + | - | + | - |
|  | 3.7 | 2.0 | 4.0 | 2.3 | 3.7 | 2.0 | 3.4 | 4.9 | 0.5 | 2.3 | 0.5 | 3.4 |

**Data Table 4: Figure 3C**

| + cofactor | | | |  |  | - cofactor | | | |
| --- | --- | --- | --- | --- | --- | --- | --- | --- | --- |
| WT | *tph-1* | *nlp-3* | *nlp-3; tph-1* |  |  | WT | *tph-1* | *nlp-3* | *nlp-3; tph-1* |
| 3 | 9 | 3 | 0 |  |  | 0 | 0 | 0 | 0 |
| 7 | 12 | 0 | 0 |  |  | 0 | 0 | 0 | 0 |
| 10 | 8 | 0 | 0 |  |  | 0 | 0 | 0 | 0 |
| 5 | 5 | 0 | 0 |  |  | 0 | 0 | 0 | 0 |
| 7 | 8 | 0 | 0 |  |  | 0 | 0 | 0 | 0 |
| 7 | 8 | 1 | 0 |  |  | 0 | 0 | 0 | 0 |
| 7 | 3 | 0 | 0 |  |  | 0 | 2 | 0 | 0 |
| 5 | 5 | 1 | 0 |  |  | 0 | 0 | 0 | 0 |
| 5 | 7 | 3 | 0 |  |  | 0 | 0 | 0 | 0 |
| 6 | 7 | 2 | 0 |  |  | 0 | 0 | 0 | 0 |
| 9 | 9 | 8 | 0 |  |  | 0 | 0 | 0 | 0 |
| 9 | 8 | 2 | 0 |  |  | 0 | 0 | 0 | 0 |
| 7 | 4 | 4 | 0 |  |  | 0 | 0 | 0 | 0 |
| 6 | 4 | 3 | 0 |  |  | 0 | 0 | 0 | 0 |
| 7 | 8 | 1 | 0 |  |  | 0 | 0 | 0 | 0 |
| 5 | 2 | 0 | 0 |  |  | 0 | 0 | 0 | 0 |
| 14 | 7 | 2 | 0 |  |  | 0 | 0 | 0 | 0 |
| 5 | 10 | 2 | 0 |  |  | 0 | 0 | 0 | 0 |
| 7 | 6 | 0 | 0 |  |  | 0 | 0 | 0 | 0 |
| 3 | 4 | 5 | 0 |  |  | 0 | 1 | 0 | 0 |

**Data Table 5: Figure 6A**

| M9 only | | | |  | | | M9 + serotonin | | |
| --- | --- | --- | --- | --- | --- | --- | --- | --- | --- |
| WT | *nlp-3* | *ser-1* |  | |  | WT | | *nlp-3* | *ser-1* |
| 5 | 0 | 2 |  | |  | 12 | | 12 | 0 |
| 2 | 0 | 1 |  | |  | 12 | | 11 | 0 |
| 3 | 0 | 2 |  | |  | 15 | | 8 | 0 |
| 6 | 0 | 2 |  | |  | 5 | | 1 | 7 |
| 6 | 0 | 5 |  | |  | 10 | | 5 | 0 |
| 6 | 0 | 0 |  | |  | 15 | | 10 | 1 |
| 1 | 0 | 3 |  | |  | 18 | | 11 | 0 |
| 3 | 0 | 4 |  | |  | 0 | | 4 | 2 |
| 1 | 2 | 0 |  | |  | 11 | | 10 | 0 |
| 2 | 2 | 0 |  | |  | 0 | | 12 | 1 |
| 4 | 0 | 1 |  | |  | 0 | | 10 | 0 |
| 7 | 1 | 0 |  | |  | 9 | | 3 | 0 |
| 4 | 0 | 4 |  | |  | 9 | | 17 | 0 |
| 0 | 1 | 4 |  | |  | 10 | | 13 | 4 |
| 2 | 0 | 0 |  | |  | 17 | | 12 | 4 |
| 5 | 0 | 8 |  | |  | 0 | | 6 | 2 |
| 5 | 0 | 7 |  | |  | 8 | | 16 | 5 |
| 2 | 0 | 2 |  | |  | 12 | | 15 | 13 |
| 1 | 1 | 2 |  | |  | 10 | | 16 | 0 |
| 3 | 0 | 0 |  | |  | 5 | | 7 | 1 |
| 10 | 0 | 4 |  | |  | 19 | | 12 | 9 |
| 0 | 0 | 3 |  | |  | 8 | | 7 | 0 |
| 2 | 0 | 0 |  | |  | 13 | | 11 | 1 |
| 0 | 0 | 0 |  | |  | 8 | | 13 | 2 |
| 6 | 2 | 6 |  | |  | 9 | | 7 | 3 |
| 1 | 0 | 1 |  | |  | 14 | | 8 | 2 |
| 1 | 0 | 3 |  | |  | 12 | | 4 | 6 |
| 8 | 2 | 0 |  | |  | 17 | | 14 | 1 |
| 0 | 0 | 0 |  | |  | 12 | | 23 | 0 |
| 0 | 0 | 3 |  | |  | 13 | | 8 | 1 |

**Data Table 6: Figure 6B**

|  | **Control** | | ***nlp-3 OX*** | | **Control in *tph-1*** | | ***nlp-3 OX* in *tph-1*** | |
| --- | --- | --- | --- | --- | --- | --- | --- | --- |
| **Number of early stage eggs (out of 250)** | 26 | | 226 | | 2 | | 185 | |
|  |  |  |  |  |  |  |  |  |
| **% Early Stage Eggs** | 10.4 | | 90.4 | | 0.80 | | 74.0 | |
|  |  |  |  |  |  |  |  |  |
| **95% Confidence Intervals** | + | - | + | - | + | - | + | - |
|  | 4.4 | 3.2 | 3.1 | 4.3 | 2.1 | 0.7 | 5.0 | 5.8 |
